# Population-level behavioral and structural drivers of COVID-19 vaccine uptake in the US

**DOI:** 10.1371/journal.pcbi.1013988

**Published:** 2026-07-20

**Authors:** Ran Xu, Navid Ghaffarzadegan, Gaofei Zhang, Goshi Aoki, Hazhir Rahmandad

**Affiliations:** 1 Department of Allied Health Sciences, University of Connecticut, Storrs, Connecticut, United States of America; 2 Department of Industrial and Systems Engineering, Virginia Tech, Alexandria, Virginia, United States of America; 3 Sloan School of Management, Massachusetts Institute of Technology, Cambridge, Massachusetts, United States of America; University of Zaragoza: Universidad de Zaragoza, SPAIN

## Abstract

Substantial variation in COVID-19 vaccination trends across U.S. communities raises key questions about the factors that shape vaccination uptake. We develop a dynamic simulation model that captures population-driven feedback processes, from social influence and uptake heterogeneity to fear of infection, responses to vaccine availability, and capacity constraints. To estimate behavioral parameters, we derive an ordinary differential equation (ODE)-informed Tobit regression framework and apply it to weekly vaccination data from all 50 US states and the District of Columbia during 2021–2022. The resulting estimates provide a robust empirical foundation for model calibration. The calibrated model reproduces observed regional vaccination trajectories and reveals three distinct phases of uptake: an early phase in which capacity and eligibility constraints bind on the underlying demand, a middle phase in which behavioral responses to changing COVID-19 incidence shape the fluctuations in uptake, with rising case numbers stimulating additional vaccination; and a late phase shaped by saturation dynamics. Finally, we document substantial regional heterogeneity in responsiveness to infection risk and the overall tendency to vaccinate when capacity permits.

## Author summary

How does COVID-19 vaccination uptake change over time, and what forces drive the vaccination uptake? We set out to understand what mechanisms shaped the COVID-19 vaccination rates and how these mechanisms evolved across the 2021–2022 rollout in the United States. We built a mathematical model that treats vaccination as a behavior that evolves in response to how many people are already vaccinated, how severe the local outbreak appears, and whether vaccines are accessible. Using weekly vaccination records from all 50 states and Washington, D.C., we estimated the strength of each force for every state. We found that our simple model can reproduce the observed vaccination trajectories, and vaccination unfolded in three phases: an early period when limited supplies constrained uptake, a middle period when rising case numbers prompted more people to get vaccinated, and a later period when uptake slowed as the most willing recipients had already been reached. States also differed substantially in how strongly residents responded to infection risk and in their overall tendency to seek vaccination when doses were available.

## Introduction

Vaccination has been one of the most effective public health interventions for controlling infectious diseases, including respiratory diseases such as COVID-19 and influenza, substantially reducing severe illness, hospitalization, and mortality [1–3]. However, in many countries—including the United States—vaccination uptake has stalled or declined, raising concerns about long-term health outcomes and population-level immunity [4–6]. In the United States, declining routine childhood immunization in recent birth cohorts [6] and large measles outbreaks concentrated in communities with low measles-mumps-rubella coverage [4,7,8] illustrate how localized gaps can translate into renewed epidemic risk. Understanding why vaccination uptake stagnates or diverges across communities is therefore not only of theoretical interest, but also has direct implications for epidemic control and health system resilience. In this study, we focus on COVID-19 as a recent and consequential case of large-scale vaccination deployment.

Despite extensive empirical study, the determinants of COVID-19 vaccination—and the policies most effective at increasing coverage—remain only partially understood [9–11]. Substantial variation in vaccination trends across U.S. communities raises fundamental questions about what drives uptake, when those drivers matter, and how they interact over time. Common explanations emphasize relatively stable compositional and attitudinal correlates, including political ideology [12,13], religion [14], trust in government [15], education [16], and sociodemographic characteristics [15–17]. While important, these factors often explain only a fraction of the observed heterogeneity and are typically treated as time-invariant over the course of an epidemic [12,18]. Even within the same political or ideological group, communities can exhibit markedly different vaccination trajectories, suggesting that additional behavioral, social, and contextual factors play an important role [18].

Beyond these individual-level correlates, community vaccination trajectories also reflect structural and contextual conditions that shape both access and demand. Early in the U.S. COVID-19 rollout, eligibility rules, supply, and delivery capacity constrained uptake, with later phases increasingly characterized by demand limitations and persistent inequities [19,20]. County-level analyses document systematic disparities by social vulnerability and urbanicity [21] and by rural–urban status [22], consistent with the role of transportation barriers, provider availability, and local health system capacity. Community networks and institutions may further amplify or dampen uptake: social capital has been associated with higher vaccination in some settings [23], yet can interact with partisan sorting to reinforce hesitancy in polarized communities [24]. Together, these findings point to vaccination uptake as an emergent community outcome shaped by the interplay of supply constraints, access, and socially mediated demand.

**Fig 1:**
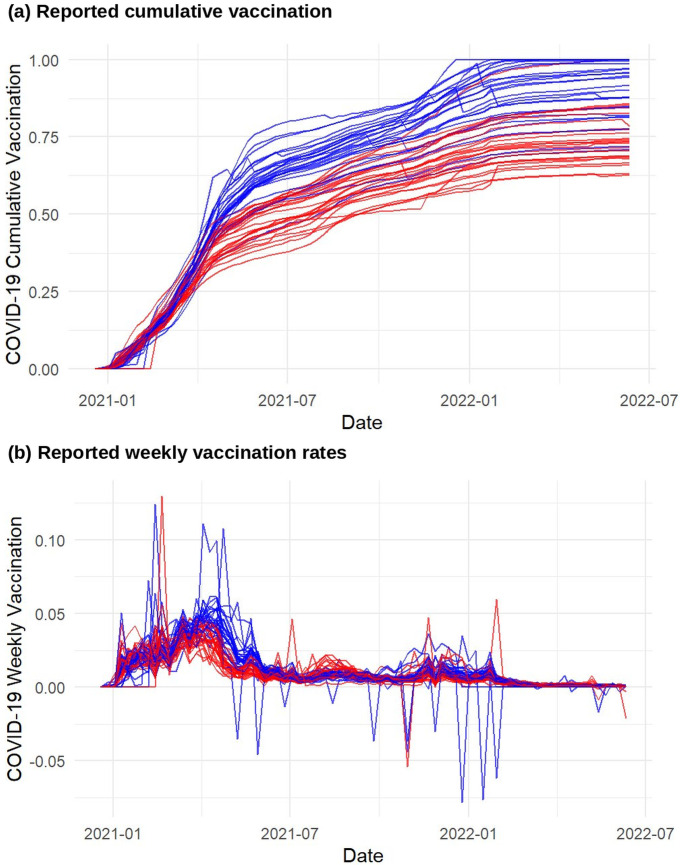
Vaccination dynamics across U.S. states. **(A)** Reported cumulative COVID-19 vaccination first-dose coverage in the age 5+ population over time. **(B)** Reported weekly COVID-19 vaccination first-dose coverage rates (fraction of total age 5+ population) over the same period, for all 50 U.S. states and Washington, D.C. Blue (vs. red) lines correspond to states with a Democratic (vs. Republican) political affiliation in 2020 presidential election. The drop in cumulative vaccination and the negative values in weekly vaccination rates are due to reporting errors and corrections in the raw data.

Most existing models and empirical analyses focus on explaining differences in final vaccination coverage, rather than the dynamic processes through which vaccination uptake unfolds over time (e.g., [13]). They therefore provide limited insight into the adoption-rate differences that determine whether vaccination occurs early enough to substantially reduce infections and deaths. Fig 1 illustrates this dynamic heterogeneity by showing state-level trends in COVID-19 vaccination across the United States. While cumulative vaccination levels differ by state—partly aligning with 2020 presidential election results—there is substantial variation within each political group, especially in early uptake rates. Moreover, weekly vaccination trajectories are not easily explained by political orientation alone, highlighting the importance of modeling vaccination dynamics rather than only final outcomes.

Capturing vaccination uptake dynamics requires models that explicitly represent how population behavior evolves in response to epidemic conditions, policy signals, and social context. Over the past decade—particularly following the COVID-19 pandemic—there has been growing attention to incorporating human behavioral change into epidemiological models [25–27]. However, the vast majority of this work has focused on modeling non-pharmaceutical interventions (NPIs), such as masking, mobility change, and business closures, as responses to changing risk perceptions [28–33], with relatively few studies treating vaccination itself as a behavioral response influenced by the evolving state of the epidemic [34,35].

Health behavior research suggests that several key determinants of vaccination are inherently dynamic. Perceived susceptibility and severity, perceived vaccine benefits and safety, and cues-to-action can change as incidence rises or falls and as new information spreads; meta-analytic evidence links risk perception to vaccination behavior [36]. In addition, psychological antecedents such as confidence, complacency, and constraints are measurable constructs that can vary across communities and over time [37]. The information environment can also shift rapidly: experimental evidence shows that exposure to online COVID-19 vaccine misinformation reduces intent to vaccinate [38], suggesting a mechanism through which epidemic trends, media narratives, and social influence can jointly generate turning points, plateaus, or reversals in uptake. Yet many community-level studies incorporate such factors only indirectly (e.g., as fixed covariates or unobserved heterogeneity), leaving a gap in mechanistic explanations of vaccination dynamics.

In this paper, we develop a dynamic simulation model that captures population-driven feedback processes shaping vaccination uptake over time. The model incorporates behavioral responses to perceived infection risk, vaccine availability, and policy changes, including eligibility expansion and capacity constraints. We treat infection cases as exogenous and use observed case data as an input capturing the epidemiological conditions faced by individuals. Accordingly, the model should be interpreted as a reduced-form representation of risk response, in the form of vaccination uptake, rather than a fully coupled epidemic–behavior system. By focusing on uptake dynamics rather than final coverage alone, our framework provides insight into the mechanisms that generate divergent vaccination trajectories across regions.

## Methods

### Data

Weekly COVID-19 vaccination data were obtained from the Centers for Disease Control and Prevention (CDC) [39]. We focused on the percentage of the population aged 5 and older who received at least one vaccine dose in each U.S. state and Washington, D.C. Weekly COVID-19 case data (per 100,000 population) for all U.S. states were obtained from the CDC COVID-19 Data Tracker. Case counts were log-transformed to normalize the distribution and satisfy the assumptions of linear regression models. Data on vaccine-eligible age groups were compiled for all 50 U.S. states and Washington, D.C. through comprehensive review of primary government sources. These included state health department eligibility announcements, press releases and executive orders, official bulletins, and local public health communications—totaling over 100 documents that enabled reconstruction of each state’s vaccine eligibility timeline across demographic groups. The detailed coding procedure and resulting state-week eligibility series are described in S2 File. This analysis is limited to the United States over the period from December 19, 2020, to June 17, 2022. This timeframe was selected to begin immediately after COVID-19 vaccines became available and to end when most states had reached vaccination equilibrium.

### Modeling

Our approach is to combine mechanistic simulation with statistical estimation. A simple simulation model captures endogenous vaccination dynamics in each U.S. state and the District of Columbia, informs statistical model specification, and later can be used for simulating counterfactuals.

**Fig 2:**
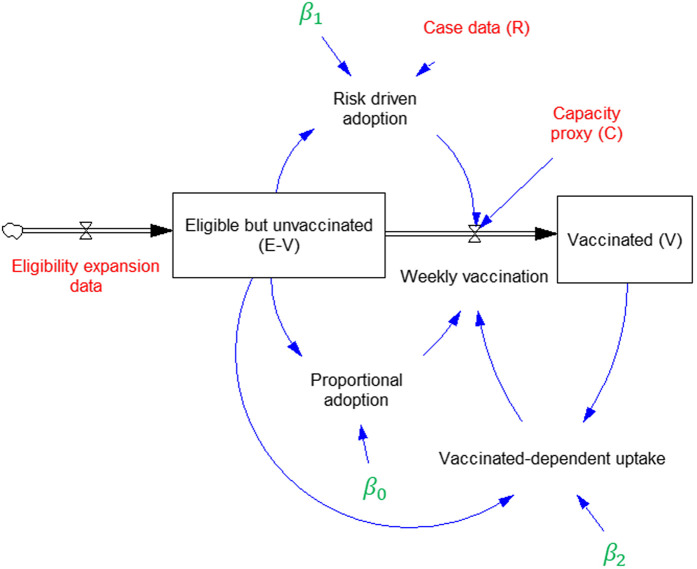
A conceptual model of vaccination uptake. This model adapts the Bass diffusion framework to describe vaccination as a dynamic process shaped by vaccine eligibility, vaccination capacity, and willingness to vaccinate upon availability, along with perceived infection risk and coverage-dependent uptake. Exogenous data inputs are shown in red, and parameters estimated from the regression model are shown in green.

Vaccination uptake in our setting exhibits a key structural feature common to diffusion processes: the rate of new adoption depends on both external drivers and the cumulative number of prior adopters. The latter effect arises naturally through feedback mechanisms such as social influence and information diffusion, which tend to increase uptake, as well as mechanisms that dampen vaccination over time: free-riding, declining perceived risk as population-level immunity increases, and individual sorting where vaccine enthusiasts vaccinate first. The Bass diffusion framework [40] provides a parsimonious way to capture this combination of exogenous and endogenous effects in a reduced-form representation. Similar feedback structures have been used to represent behavioral responses affecting non-pharmaceutical intervention (NPI) compliance (e.g., [33,41]) as well as vaccination uptake [42]. These formulations often yield dynamics that are closely related in structure to diffusion-type models, even when not explicitly framed in those terms. Our approach is particularly closely connected to Epstein et al.’s formulation, in which vaccination behavior responds to perceived risk and population-level dynamics [42]. We differ in adopting a reduced-form specification that is directly amenable to empirical estimation in a regression framework across states. A conceptual representation of the model is shown in Fig 2.

The model tracks the movement of people across two stocks: from eligible but unvaccinated to those already vaccinated. In a model with a weekly time unit, the flow from eligible to vaccinated represents the weekly vaccination increment. A standard component of diffusion models is proportional adoption, where a fraction of potential adopters (eligible but unvaccinated) gets vaccinated each week. This fraction ($\beta_{0}$) captures the baseline propensity to vaccinate among eligible individuals, conditional on vaccine availability.

We extend this baseline model in three ways. First, we included an exogenous risk indicator (weekly COVID-19 cases per 100,000 population; log-transformed) to model how vaccination rates respond to perceived risk, as prior studies have shown that behavior (e.g., mobility) is affected by external risk [43]. Second, we incorporate vaccination capacity, which has been shown to limit the vaccination rate, particularly during the early rollout [20]. Third, we included age-specific vaccination eligibility, reflecting the phased expansion of vaccine eligibility from older and high-risk populations to younger, general populations [19].

Mathematically, let $V_{t}\in [0,1]$ denote the fraction of the total population aged 5+ in a state vaccinated at time *t*, and $E_{t}$ the fraction who currently are eligible for vaccina*t*ion. Let $dV_{t}$ represent new vaccinations. We model vaccination dynamics as:


$$\begin{array}{c} dV_{t}=\min\;\left(C_{t},\;(E_{t}-V_{t})(\beta_{0}+\beta_{1}R_{t}+\beta_{2}V_{t})\right)\;dt, \end{array}$$ (1)

where $C_{t}$ is the weekly vaccination capacity, which places an upper bound on weekly vaccinations. The terms on the right-hand side capture: a baseline proportional adoption $\beta_{0}(E_{t}-V_{t})$, adoption driven by external risks $\beta_{1}(E_{t}-V_{t})R_{t}$, and an endogenous adoption driven by vaccination coverage $\beta_{2}(E_{t}-V_{t})V_{t}$. Here, $\beta_{0}$, $\beta_{1}$, and $\beta_{2}$ represent the effect of proportional adoption, risk-driven adoption, and vaccinated-dependent uptake, respectively. The risk term $R_{t}$ is the weekly log-transformed COVID-19 cases per 100,000 population in each state, while $\beta_{2}$ captures net effect of current vaccinated such as word-of-mouth effects, free riding, and draining of those most interested in vaccines.

For statistical estimation, we use a discrete weekly formulation. Dividing both sides by the unvaccinated fraction $\left(E_{t-1}-V_{t-1}\right)$, we have


$$\begin{array}{c} \frac{\Delta V_{t}}{E_{t-1}-V_{t-1}}=\min\;\left(\frac{C_{t}}{E_{t-1}-V_{t-1}},\;\beta_{0}+\beta_{1}R_{t-1}+\beta_{2}V_{t-1}\right), \end{array}$$ (2)

where $\Delta V_{t}=V_{t}-V_{t-1}$ is the observed weekly vaccination increment. Weekly capacity denotes the maximum amount of vaccines each state can administer in a specific week, which is affected by various factors such as the number of doses allocated and delivered, operational administration sites, staffing and scheduling. There is no reliable and consistent data at the state-week level for vaccination capacity. We therefore used an outcome-driven approach to estimate weekly capacity as the maximum observed increment up to time *t*:


$$\begin{array}{c} C_{t}=\max\{\Delta V_{1},\Delta V_{2},\ldots ,\Delta V_{t}\}. \end{array}$$ (3)

This formulation reflects the intuition that the highest weekly uptake realized up to any point represents the ceiling of what the delivery system was able to process under the most favorable conditions observed so far. It is motivated by several features of the early U.S. COVID-19 vaccination rollout. First, vaccine supply and administration infrastructure expanded sharply over the first several months and the distribution network remained available once built [44]. Second, studies showed that early vaccination rates were consistently constrained by supply shipments, allocation quotas, and the number of available administration sites rather than by a lack of willing recipients [20]. Under these conditions, the historical maximum provides a natural, data-derived upper bound: weeks in which observed uptake fell well below this maximum are interpreted as capacity-unconstrained, while weeks at or near the maximum are treated as capacity-constrained.

Because the dependent variable $\Delta V_{t}/(E_{t-1}-V_{t-1})$ is censored above by capacity, the parameters $\beta_{0}$, $\beta_{1}$, and $\beta_{2}$ are estimated using an upper-censored Tobit regression for each state:


$$\begin{array}{c} y_{t}=\min\;\left(\frac{C_{t}}{E_{t-1}-V_{t-1}},\;\beta_{0}+\beta_{1}R_{t-1}+\beta_{2}V_{t-1}\right), \end{array}$$ (4)

where $y_{t}=\Delta V_{t}/(E_{t-1}-V_{t-1})$. The upper-censored Tobit model is appropriate here because the dependent variable $y_{t}$ is structurally censored from above by the capacity ratio $C_{t}/(E_{t-1}-V_{t-1})$: when capacity constraint is binding, observed uptake equals the capacity ceiling regardless of the underlying behavioral propensity, so OLS would systematically underestimate the underlying parameters. The Tobit likelihood corrects for this by treating censored and uncensored weeks differently: uncensored weeks contribute through estimating the exact density function in the absence of the ceiling, whereas censored weeks contribute through estimating the probability of exceeding the ceiling. While both observations contribute information to the estimation process, the behavioral parameters $\beta_{0}$, $\beta_{1}$, and $\beta_{2}$ are identified heavily from weeks in which the capacity constraint was not binding and thus more closely reflect the underlying (uncensored) parameters [45]. Consistent estimation assumes the censoring threshold is correctly specified, errors are normally distributed, and the latent model (uncensored) is linear. During estimation we only used observations where $dV_{t}\geq 0$, as negative $dV_{t}$ can emerge due to reporting errors and corrections that do not reflect the true behavioral mechanism. We note that while this choice should result in more reliable estimates of the behavioral parameters, it could also result in a worse predictive fit of the cumulative vaccination $V_{t}$ if there are frequent or large corrections.

The estimated coefficients are then used in equation (1) to simulate vaccination dynamics with endogenous (rather than data-driven) vaccination flow changing the stocks of $\left(E_{t}-V_{t}\right)$ and $V_{t}$. National-level predictions are obtained by summing state-level predictions weighted by population.

To account for model uncertainty, for each state we generated 1,000 sets of $\beta_{0}$, $\beta_{1}$, and $\beta_{2}$ from a multivariate normal distribution defined by the estimated mean and variance-covariance from the Tobit model. We report the 2.5–97.5 percentile range of simulated weekly and cumulative vaccination as the 95% confidence interval. Model fit was assessed by R-squared comparing the median simulated values with observed vaccination rates and cumulative vaccinations.

As robustness checks, we first verified the significance and direction of $\beta_{0}$, $\beta_{1}$, and $\beta_{2}$ by including additional covariates in each state’s Tobit model, including four lags of the dependent variable, a quadratic time trend, and seasonal effects. Second, we evaluated the contribution of risk-driven adoption, eligibility, and vaccination capacity by comparing the R-squared (from the statistical model) of the weekly vaccination rates with and without each component. Third, we tested the inclusion of dynamic proportional adoption rate by adding a quadratic time trend or death risk, calculated as the average age-specific death risk [46] (weighted by the currently eligible but unvaccinated population), in the statistical models. In addition, we tested using the rolling averages of the past 3 weeks’ capacity constraint as an alternative formulation to smooth out the influence of single spikes; we also tested constraining the predicted weekly vaccinations $dV_{t}\geq 0$, as negative increments can arise due to reporting corrections, which were excluded from the estimation. Finally, we conducted an out-of-sample validation test by estimating model parameters using only the first 60% of the data (through week 46) for all states. After parameters are estimated, we simulate the model forward to evaluate its ability to reproduce the remaining 40% of the observations.

## Results

**Fig 3:**
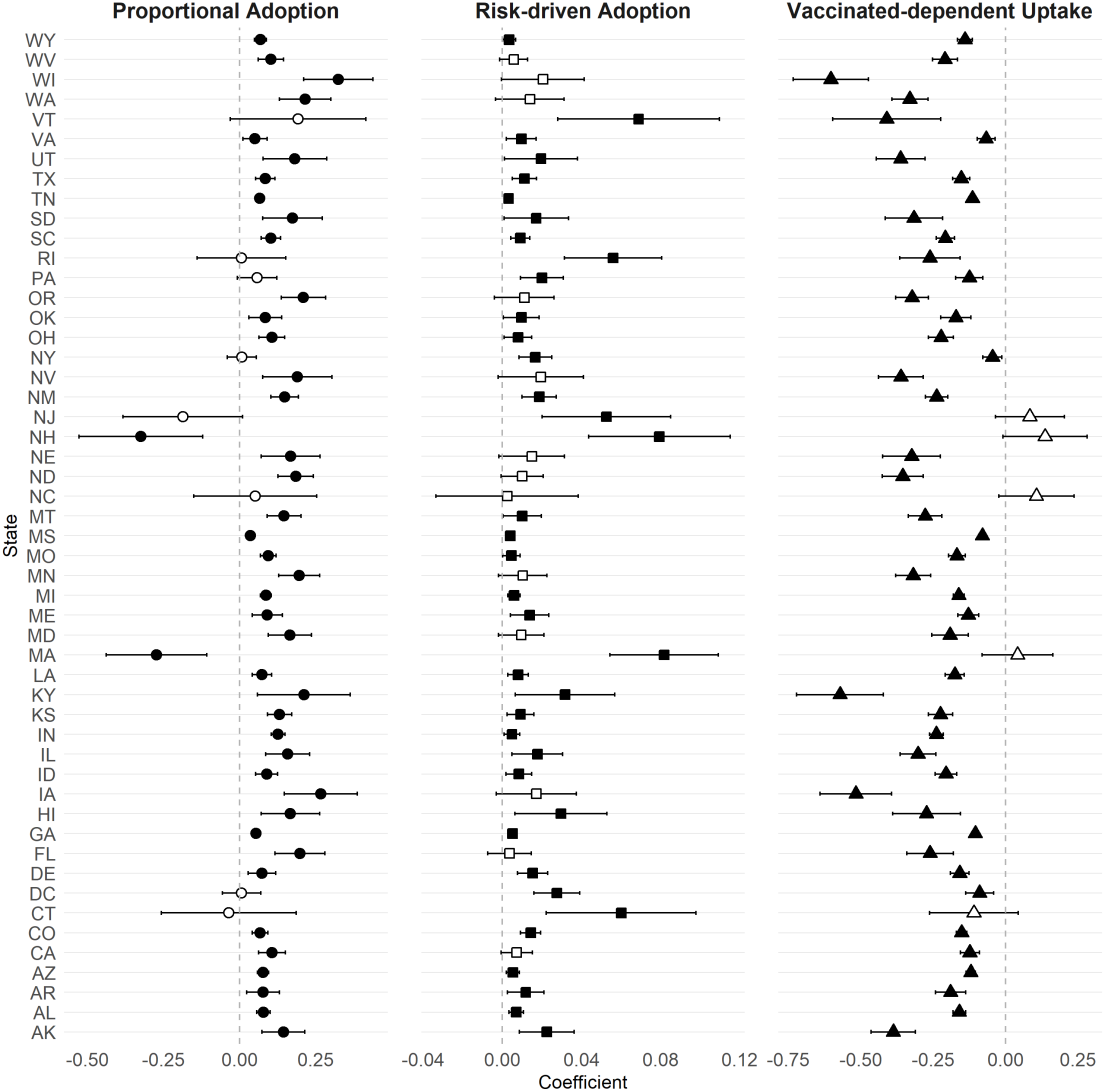
Coefficients by State: Coefficients along with 95% CI for each state and Washington, D.C. from the upper-censored tobit model on effects of proportional adoption $\beta_{0}$, risk-driven adoption $\beta_{1}$, and vaccinated-dependent uptake *β*_2_.

The coefficients and 95% confidence intervals for the effects of proportional adoption ($\beta_{0}$), risk-driven adoption (cases; $\beta_{1}$), and vaccinated-dependent uptake ($\beta_{2}$) from the upper-censored Tobit model for each state are shown in Fig 3. As illustrated, proportional adoption rates ($\beta_{0}$) were positive and significant for most states (mean = 0.096, median = 0.095, IQR [0.067, 0.168]), indicating a positive baseline vaccination rate proportional to the currently eligible but unvaccinated population. Risk-driven adoption ($\beta_{1}$) effects were also largely positive and significant in most states (mean = 0.019, median = 0.011, IQR [0.008, 0.019]), reflecting a modest but positive increase in vaccination rates in response to exogenous COVID-19 risk. Where $\beta_{0}$ was estimated to be negative (e.g., Massachusetts and New Hampshire), $\beta_{1}$ was estimated to be much larger, highlighting the partial collinearity of the baseline and risk-driven vaccination, where given continued COVID-19 infections, one could partially compensate for the other.

Effects of vaccinated-dependent uptake ($\beta_{2}$) were negative and significant in most states (mean = −0.209, median = −0.192, IQR [−0.310, −0.124]), indicating that higher existing coverage slows the weekly vaccination rate among those still eligible. Ex-ante, this effect could go either way as a positive value would highlight social influence as important to vaccination, whereas a negative effect underlines two effects. First is a selection mechanism such that those more eager to vaccinate get vaccinated earlier, leaving the reluctant people in the unvaccinated but eligible pool. Second one is more mechanical - with positive effects in proportional adoption and risk-driven adoption, there needs to be a negative effect to drive predicted vaccination rates to zero before saturation. The overall negative vaccinated-dependent uptake effect suggests that the selection mechanisms may be stronger than the social influence pathway.

To assess potential omitted variable bias, we reran the upper-censored Tobit model for each state including additional covariates - four lags of the dependent variable, a quadratic time trend, and seasonality. The results largely confirmed the direction and significance of the original coefficients, although the effect of risk/cases ($\beta_{1}$) was attenuated in some states, decreasing from 38 states with significant positive effects to 22 states (see coefficient distribution in Fig A1 in S1 File).

**Fig 4:**
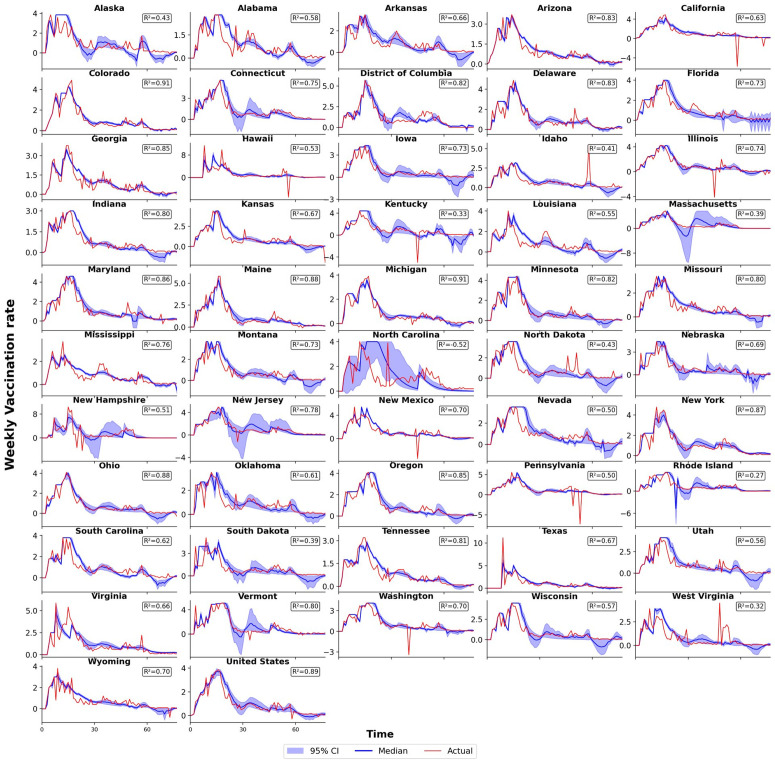
Weekly Vaccination Rate by State Over Time: Time-series plots of the weekly vaccination rates (percentage change per week) for each U.S. state and Washington, D.C. The solid blue line shows the median simulated trajectory with the shaded area showing 95-percent confidence interval. The red line depicts observed weekly vaccination data over time. The bottom-right panel displays the population-weighted aggregate for the United States.

**Fig 5:**
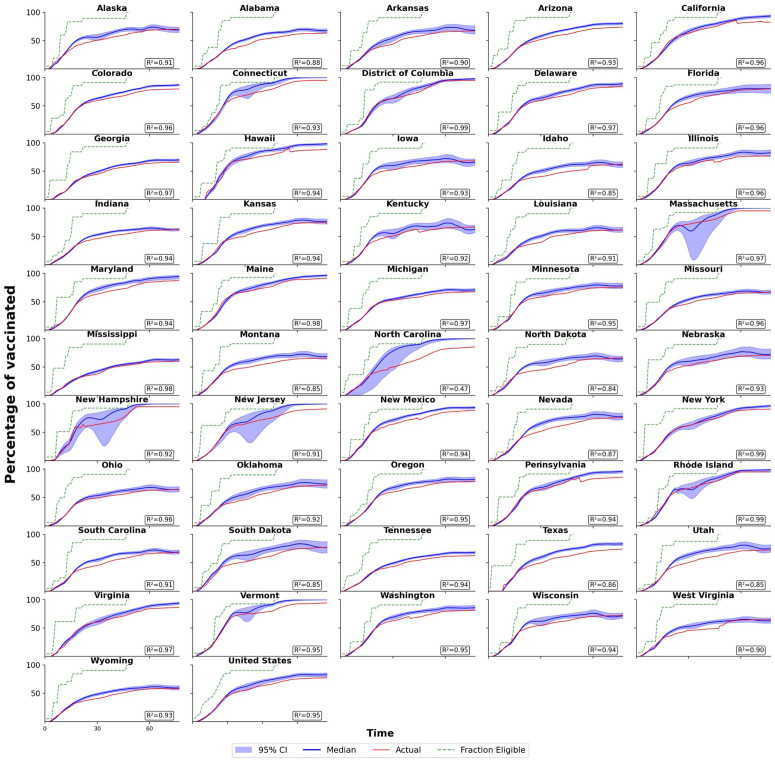
Total Vaccination Coverage by State Over Time: Time-series plots of the cumulative percentage of the vaccinated population for each U.S. state. The solid blue line shows the median simulated trajectory with the shaded area showing the 95-percent confidence interval. The red line depicts observed vaccination data, and the green dashed line depicts the vaccine-eligible fraction over time. The bottom-right panel displays the population-weighted aggregate for the United States.

These coefficients along with the uncertainty estimation from the statistical models were fed into the simulation model, and Fig 4 presents the simulated trends for weekly vaccination for each region and the United States, along with the 95 percent confidence intervals. The results showed that our simple dynamic model fits most states reasonably well (R-squared based on the median simulated values: mean = 0.64, median = 0.70, IQR [0.54, 0.81]) with a few exceptions (e.g., North Carolina) and those with large CIs (e.g., Massachusetts). The results for total vaccination coverage were similar (R-squared based on median simulated values of weekly vaccination: mean = 0.92, median = 0.94, IQR [0.91, 0.96]). Figure 5 presents the simulated total vaccination coverage for each state and the United States, along with the 95 percent confidence interval, as well as the fraction of vaccine-eligible population over time (green dotted lines). There is a minor but consistent discrepancy between the simulations and data: in many states, right after the main peak, vaccination rates drop faster than what our models predict. As a result, the observed total vaccination coverage stays slightly lower than the model prediction after that peak. This may partially be due to rapid vaccination among those most interested in vaccines, which leaves a more reluctant pool of unvaccinated. While our model accounts for this effect through a linear effect of vaccinated-dependent uptake, the actual effect is likely nonlinear and we would need more complex specifications to improve the fit.

Several additional analyses and robustness checks were performed. We tested how our model performs without each of the three key components that extend the basic diffusion model, namely risk-driven adoption, eligibility and vaccination capacity. Table 1 compares the R-squared of the weekly vaccination rate for each state from our main statistical model with the one missing each component. It shows that the mean R-squared dropped from 71.7% to 59.3%, 63.8%, and 68.7% if we ignore vaccination capacity, eligibility and risk respectively. The smaller decrease in R-squared when ignoring risks is consistent with the weak but positive case effects we observed in most states. The more significant decrease in R-squared when ignoring vaccination capacity and eligibility were consistent with the fact that a) in most states the observed vaccination rates in the first 7–12 weeks were upper censored, reflecting the limited availability of vaccine especially in the early periods, and b) vaccination eligibility of different age groups significantly changed over time (see Fig 5) which inevitably impacted who can be vaccinated and thus the vaccination rate. Then we tested whether our model performance improves by including additional components in the statistical model. A time-varying (quadratic time trend) proportional adoption rate (instead of a constant) enhanced fit (mean R-squared = 74.2%, IQR [66.7%, 85.6%]), whereas inclusion of death risk (weighted by each age group in the currently eligible but unvaccinated population) had a limited effect (mean R-squared = 71.8%, IQR [64.9%, 83.1%]). We also tested using alternative formulations of capacity constraint by using the rolling averages of the past 3 weeks’ capacity to smooth out the single early spike (no improvement; mean R-squared = 69.9%, IQR [60.3%, 80.4%]), and constrained the predicted vaccination rate to be non-negative (mean R-squared = 73.7%, IQR [66.2%, 84.8%]) as sometimes our model predictions gave negative predicted values of $dV_{t}$ but mechanistically $dV_{t}$ cannot go below 0.

**Table 1:** Comparing model fit of the weekly vaccination rate between the main statistical model and the one missing a key component.

R-squared	Mean	Median	25th Percentile	75th Percentile
Baseline	71.7%	75.0%	64.2%	82.8%
Ignore Capacity	59.3%	64.8%	47.6%	74.1%
Ignore Eligibility	63.8%	72.8%	58.6%	80.1%
Ignore Risk/Case	68.7%	71.5%	62.2%	80.1%

To assess the robustness of the estimated behavioral mechanisms beyond in-sample fit, we performed an out-of-sample validation exercise for each U.S. state and Washington, D.C. Model parameters were estimated using only the first 60% of each vaccination time series (weeks 0–46). The estimated parameters were then held fixed, and the model was simulated over the full study horizon, using observed infection case data as an exogenous input. Model performance was evaluated on the held-out period (weeks 47–77) by comparing simulated and observed vaccination uptake trajectories. Across the 50 states and Washington, D.C., the average correlation between simulated and observed uptake during the hold-out period was 0.55 (SD = 0.24). For comparison, when the same time period was included in the calibration window (i.e., evaluated in-sample), the corresponding average correlation was 0.65 (SD = 0.21). This relatively small decline in correlation suggests that the model captures behavioral uptake dynamics that generalize beyond the estimation window. Detailed state-level validation results are provided in S3 File.

### Comparing the mechanisms

**Fig 6:**
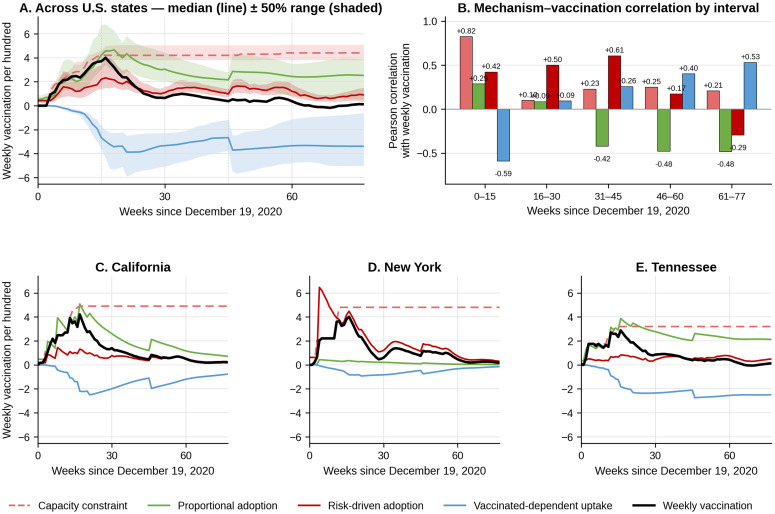
Contributions of behavioral mechanisms to vaccination dynamics. Pink indicates vaccination capacity (dashed), green indicates proportional adoption, red indicates risk-driven adoption, blue indicates vaccination-dependent uptake. **Panel A:** cross-state median (solid lines) of each model component over time, with shaded bands showing the 50% range across the 51 U.S. jurisdictions. **Panel B:** correlation between observed weekly vaccination and each of the four mechanisms within five 15-week intervals, computed across all (state, week) observations in each interval. **Panels C–E:** decomposition for three illustrative states—California, New York, and Tennessee. Subject to the capacity ceiling, the three behavioral terms sum to reproduce the observed weekly vaccination rate.

To identify what drives dynamics of vaccination uptake, we decompose simulation results of weekly vaccination into the three behavioral components specified in Equation 1—proportional adoption ($\beta_{0}$), risk-responsive behavior driven by COVID-19 incidence ($\beta_{1}$), and vaccinated-dependent uptake ($\beta_{2}$)—together with the upper bound imposed by vaccination capacity. Figure 6 presents the result in five panels. Panel A reports the cross-state median of each component over time, with shaded bands showing the 50% range across the 51 U.S. jurisdictions; these lines capture the typical *magnitude* of each mechanism’s contribution to the level of weekly vaccination. Panel B reports the correlation between observed weekly vaccination and each mechanism’s contribution to that number within five 15-week intervals, indicating which mechanism most strongly tracks point-to-point variation of vaccination at each stage. Panels C–E show the deterministic decomposition for three illustrative states of California, New York, and Tennessee (see all states in Fig A2 in S1 File). By construction, the sum of the three behavioral terms, subject to the capacity ceiling, reproduces the simulated weekly vaccination rate. Comparing them helps in understanding the magnitude of their contributions as well as the drivers that dominate in different periods of time.

Reading Panels A and B together reveals a three-phase pattern in which all four mechanisms contribute meaningfully to vaccination rates while their roles in shaping its temporal variation shift across phases. First, during the early rollout (approximately weeks 0–15), vaccination is supply-limited. The median observed and capacity curves nearly coincide in Panel A, and in Panel B the correlation between capacity and weekly vaccination reaches +0.82, reflecting that the ceiling is binding. The underlying demand in this phase is generated primarily by the constant term for proportional adoption ($\beta_{0}$), which often sets an upper bound for vaccination rates given negative estimates for $\beta_{2}$.

Second, once the capacity constraint relaxes (approximately week 16 onward), proportional adoption continues to provide the largest sustained contribution to the weekly vaccination, while risk-responsive behavior becomes the dominant driver of its weekly variation. The risk-driven correlation rises to +0.50 in weeks 16–30 and peaks at +0.61 in weeks 31–45. The state-level decompositions in Panels C–E show a similar point as fluctuations of the black and red lines follow closely.

Third, in the late phase (approximately weeks 46 onward), saturation dynamics become increasingly important. Vaccinated-dependent effect on uptake ($\beta_{2}$) grows in absolute magnitude as more people become vaccinated—contributing negatively to weekly uptake (Panel A). Net weekly vaccination is small in this phase because the substantial positive contributions of proportional and risk-driven adoption are increasingly offset by saturation.

Taken together, these patterns reveal a temporal transition in dominant mechanisms. While all four mechanisms contribute meaningfully to the dynamics of vaccine uptake, supply constraints bind in the early phase, while proportional adoption is the main force behind the demand. Incidence-driven risk responses play an important role, and primarily drive the fluctuations throughout the period, and saturation governs the diminishing rate of late-phase uptake. The magnitude and timing of each mechanism differ across states (Panels C–E), reflecting heterogeneous demographic, behavioral, and institutional contexts.

## Discussion

We studied COVID-19 vaccination trends across the 50 U.S. states and Washington D.C. during 2021–2022, with a focus on the population-level dynamics that drive weekly vaccination uptake. To this end, we adapted a simple dynamic simulation framework rooted in diffusion models to the context of vaccine adoption. The model explicitly links vaccination rates to behavioral responses, allowing uptake to adjust endogenously to perceived infection risk, social influence from previously vaccinated individuals, and evolving supply-side conditions such as eligibility expansions and capacity constraints. Rather than treating vaccination coverage as a static endpoint, our approach emphasizes the dynamic processes that generate divergent vaccination trajectories across regions.

The calibrated model closely reproduces observed vaccination trajectories across states and reveals systematic patterns in the drivers of uptake. In particular, we document substantial regional heterogeneity in both responsiveness to infection risk and baseline willingness to vaccinate when supply is available. These differences help explain the notable variation in vaccination paths observed across states, even under broadly similar national conditions. In addition, across most regions, higher cumulative vaccination levels are associated with a reduction in subsequent uptake beyond what would be expected from simple saturation alone, highlighting the importance of shifting composition of unvaccinated as the more eager get vaccinated earlier and dampen demand over time.

Examining the model’s dynamics over time further reveals three qualitatively distinct phases of vaccination uptake. The early phase is dominated by institutional and logistical factors—most notably vaccine eligibility rules and capacity constraints—those limit vaccination relative to the underlying demand generated by proportional adoption. Once the capacity ceiling relaxes, the middle phase is primarily behaviorally driven and rooted in demand fluctuations, with vaccination responding to contemporaneous changes in COVID-19 incidence. During this period, increases in reported case numbers are associated with renewed vaccination activity, highlighting the role of perceived infection risk, and attention reorientation, in sustaining uptake once supply-side barriers have largely been removed. The late phase is dominated by saturation dynamics, reflecting the changing propensity to vaccinate in the shrinking pool of unvaccinated: vaccinated-dependent uptake exerts an increasingly negative pull on weekly vaccination, offsetting the continuing positive contributions of proportional adoption and risk responses and producing the diminishing rate of late-phase uptake.

This study contributes to the epidemiological and public health modeling literature along several dimensions. First, it adds to research on the determinants of vaccination uptake [9,12–14] by explicitly modeling vaccination as a dynamic process rather than a static outcome. While much of the existing literature relies on cross-sectional or survey-based analyses to explain vaccination intentions or coverage [13,15,16], our approach captures how uptake evolves over time in response to changing epidemic conditions and institutional constraints. In particular, the model incorporates risk-driven behavioral responses to change in infection levels, a mechanism that is often difficult to identify using self-reported survey data alone. Methodologically, this distinguishes our work from prior studies by leveraging epidemic and administrative data to infer behavioral responses, rather than relying primarily on stated preferences or intentions.

Second, this work contributes to the growing literature that incorporates change in human behavior in epidemic dynamics [27–30] by estimating vaccine uptake as a form of human response to changing risks. Whereas much of this literature has focused on NPIs—such as social distancing [32,47], mobility reduction [31], or mask use [33]—as adaptive responses to disease prevalence, we examine vaccination as a behavioral response to epidemic conditions. In this respect, our results resonate with a small number of simulation-based studies that incorporate endogenous vaccination behavior [34,35,42], but extend them by providing empirical estimates of behavioral responsiveness and by documenting substantial regional heterogeneity in these mechanisms.

Third, a methodological contribution of this study lies in linking a dynamic simulation model to a formal statistical estimation framework. By expressing weekly vaccination uptake as a fraction of the eligible yet unvaccinated population, introducing a proxy for capacity constraints, and assembling a detailed, state-level dataset on vaccine eligibility over time, we are able to linearize the dynamic system and estimate behavioral parameters with uncertainty quantification. This formulation enables the use of an ODE-informed Tobit regression approach to obtain parameter estimates with confidence intervals, which are then used to calibrate the simulation model. More broadly, this approach illustrates how dynamic behavioral models can be empirically grounded using routinely collected epidemic and administrative data, helping to bridge the gap between simulation-based modeling and statistical inference.

This study has several limitations that also point to directions for future research. First, we intentionally adopt a parsimonious modeling framework and only include a few key behavioral mechanisms governing vaccination uptake. As a result, we did not include the more complex model specifications or many other dynamic factors that could influence vaccination uptake such as changes in perception of vaccination effectiveness/safety or trust in government over time, which have been shown to influence willingness to vaccinate [10,11]. We also do not model cross-state interactions, such as how vaccination dynamics or social signals in one state may influence behavior in neighboring or demographically similar states. Incorporating spatial or network-based coupling across regions would allow for separating social influence and selection dynamics which we have lumped together. Second, infection dynamics are treated as exogenous in order to focus on parameter estimation for vaccination behavior and to avoid compounding uncertainty from jointly estimating epidemic and behavioral processes. Future work could re-integrate the vaccination adoption framework into an SEIR-type model to endogenously simulate epidemic trajectories alongside vaccination responses, in a coupled framework. In this sense, our approach aligns with what has been described as partial-model testing [48], as it isolates the effect of changing case incidence on vaccination decisions from a relatively more lagged feedback of vaccination on disease dynamics. Third, our model specification is necessarily aggregate and does not explicitly capture within-state heterogeneity by age, socioeconomic status, or political affiliation, all of which are known to influence vaccination decisions [17,49,50]. Similarly, while we document substantial cross-state heterogeneity in behavioral parameters such as baseline willingness to vaccinate and responsiveness to infection risk, we do not systematically link these differences to observable state characteristics. Future studies could incorporate these factors, examine the correlates of estimated behavioral parameters across states, and build models on a more detailed level. Finally, our proxy for capacity constraints is data-derived rather than independently measured and therefore cannot cleanly separate true delivery-system capacity from periods of peak demand. Our sensitivity analyses demonstrated that this capacity constraint proxy played a genuine structural role, as it was the main driver/constraint of vaccine uptake in the first 7–12 weeks, and removing it from the model resulted in meaningful deterioration of the model’s performance. Future work could explore alternative formulations and assemble data to generate a more structurally grounded capacity measure.

In summary, this study provides a data-driven, dynamic account of how population-level behavioral mechanisms—particularly vaccination responses to epidemic risk—shape uptake over time. By grounding the framework in empirical estimation, our results help clarify why vaccination trajectories varied across U.S. states despite broadly similar national conditions. We view this work as a step toward more integrated behavioral–epidemiological modeling approaches that treat vaccination uptake as a dynamic response to epidemic conditions, while balancing interpretability, empirical grounding, and policy relevance.

## Supporting information

S1 File Additional analysis. (PDF)

S2 File Reconstruction of state-level vaccine eligibility. (PDF)

S3 File Out-of-sample test results. (PDF)
