# Supplementary material for: Population-level behavioral and structural drivers of COVID-19 vaccine uptake in the US
Source: PLoS Comput Biol. 2026 Jul 20;22(7):e1013988. doi: 10.1371/journal.pcbi.1013988 (PMC13405102; doi:10.1371/journal.pcbi.1013988)
Supplement: S1 File — (PDF) [file pcbi.1013988.s001.pdf]

Appendix A: Additional analysis

Sensitivity analysis

Fig A1 presents the state-specific coefficient estimates and 95% confidence intervals from the upper-censored Tobit model after controlling for four lags of the dependent variable, seasonality, and a quadratic time trend. The directions and statistical significance of the parameter estimates are largely consistent with those in the main model.

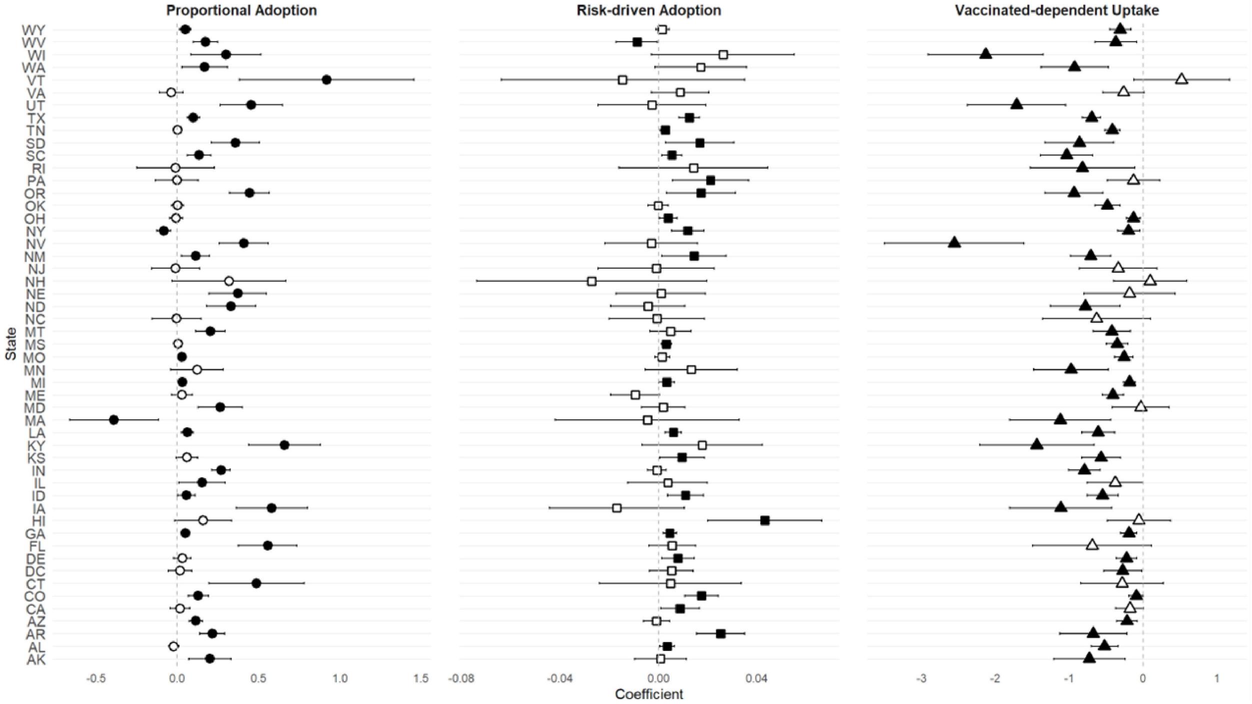

Fig A1: **Coefficients by State:** Coefficients along with 95% CI for each state from the upper-censored Tobit model on effects of proportional adoption β_0, risk-driven adoption β_1, and vaccinated-dependent uptake β_2, after controlling for 4 lags of the dependent variable, seasonality, and quadratic time trend.

State-level decompositions

Fig A2 reports the four-mechanism decomposition of weekly vaccination dynamics (Equation 1) for all 51 U.S. jurisdictions and the cross-state median, complementing the main-text analysis.

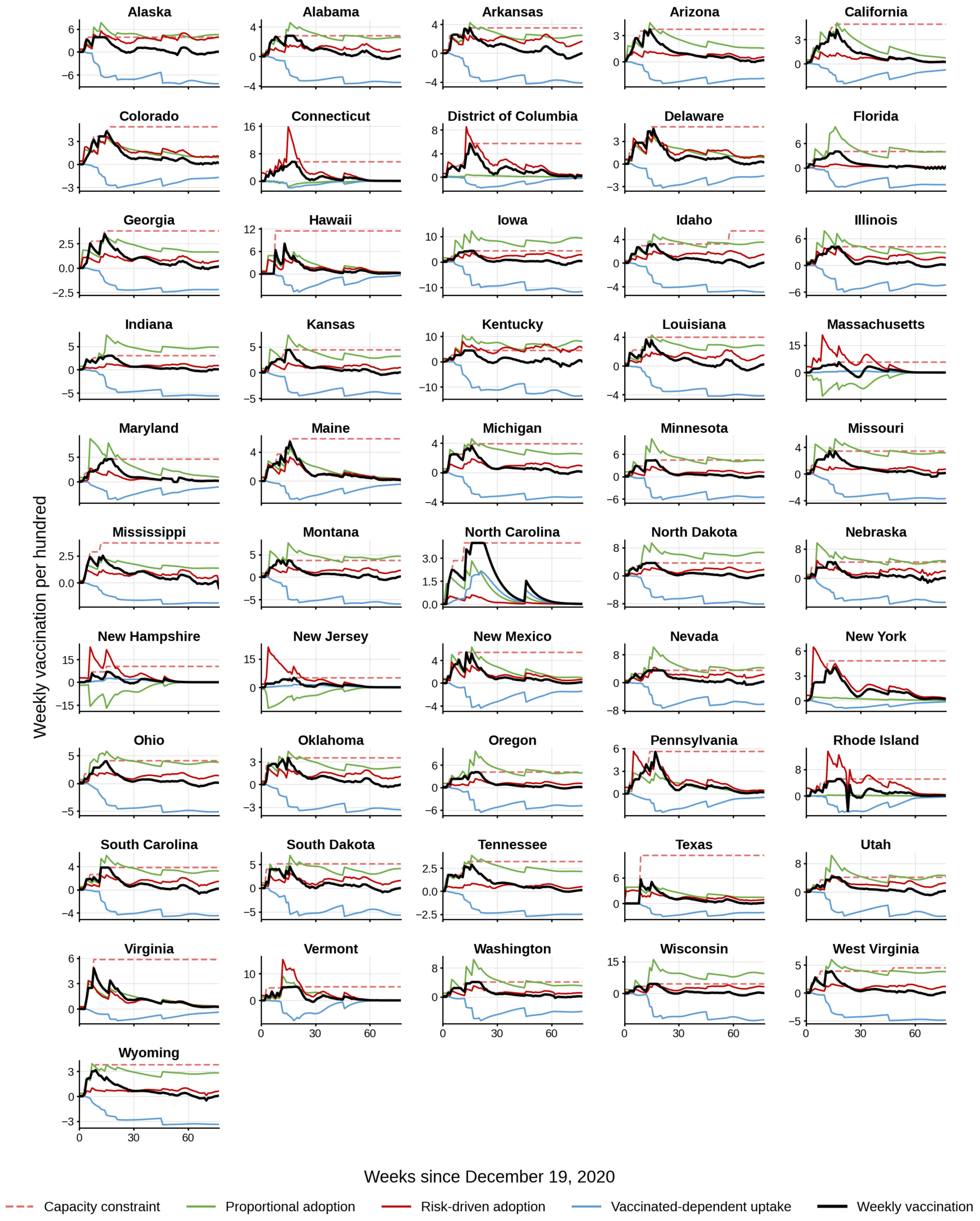

Fig A2: **State-level decomposition of weekly vaccination dynamics.** For each U.S. state, the District of Columbia, weekly vaccination per hundred (solid black) is decomposed into the three behavioral terms of Equation 1— proportional adoption (green), risk-driven adoption (red), and vaccinated-dependent uptake (blue)—together with the capacity-and-eligibility constraint (dashed red).
