# Supplementary material for: Population-level behavioral and structural drivers of COVID-19 vaccine uptake in the US
Source: PLoS Comput Biol. 2026 Jul 20;22(7):e1013988. doi: 10.1371/journal.pcbi.1013988 (PMC13405102; doi:10.1371/journal.pcbi.1013988)
Supplement: S2 File — (PDF) [file pcbi.1013988.s002.pdf]

Appendix B: Reconstruction of State-Level Vaccine Eligibility

Since vaccine eligibility varied substantially across states and over time, we reconstructed a weekly state-level eligibility series and used it as the $E_{s,t}$ input in the model. The objective of this reconstruction was to convert qualitative state policy announcements into a quantitative measure of the fraction of each state's population eligible to receive a vaccine in each week.

Source documents

The primary source was Ballotpedia's tracker "Coronavirus (COVID-19) vaccination rates and distribution plans by state" [1], which compiles dated, citation-backed records of every state-level eligibility decision from December 2020 through November 2021. For each entry we retained both the underlying narrative text and the link to the state's primary document—typically a gubernatorial press release, state department of health bulletin, or executive order. Where Ballotpedia's account was incomplete or ambiguous, we checked the primary source directly.

Event extraction and dating

We extracted discrete eligibility-decision events across all states and the District of Columbia covered in our analysis. Each event was assigned an effective date using the following rule: when an announcement preceded the actual implementation date, we coded the event to the week containing the implementation date—the week residents could begin booking appointments—rather than the announcement week. Weeks were defined as intervals beginning on Monday, with week 1 corresponding to the first U.S. administration of an EUA-authorized vaccine (December 14, 2020).

Categorical coding

Each event was tagged with one or more categories spanning four occupational and risk-based groups (health care workers, essential workers, educators, persons with high-risk health conditions) and multiple age thresholds (80+, 75+, 70+, 65+, 60+, 55+, 50+, 45+, 40+, 30+, 25+, 20+, 18+, 16+, 15+, 12+, 10+, 5+). Tags accumulate cumulatively within state: once a group is eligible, it remains eligible.

Demographic mapping

To convert the categorical coding into a population fraction, we mapped each tag to a state-specific share using publicly available data:

- **Age thresholds.** Cumulative population at or above each threshold was calculated from the Annual Estimates of the Civilian Population by Single Year of Age and Sex for the United States and States [2].

- **Health care workers.** Health care worker eligibility used the Kaiser Family Foundation estimates of health care workers with direct patient contact plus nursing facility residents [3].
- **Essential workers.** Essential-worker eligibility used the United Way NCA state-level estimates of the share of the labor force working in essential industries [4].
- **Persons with high-risk health conditions.** High-risk medical condition eligibility used a state-invariant prevalence [5].

Aggregation rule for $E_{s,t}$

For each state-week, the coded eligibility categories were converted into an eligible-population count, $N_{s,t}^{elig}$. Age-based eligibility was mapped directly to the corresponding state age group. For combined age and non-age rules, the non-age eligibility share was applied to the residual population below the relevant age threshold. For example, when eligibility included persons aged 65 or older, essential workers, and persons with high-risk conditions, we compute

$$N_{s,t}^{elig} = N_s^{65+} + N_s^{16\text{–}64} \left(\rho_s^{ess} + \rho^{cond} \right), \tag{1}$$

where N_s^{65+} is the state population aged 65 or older, $N_s^{16\text{–}64}$ is the state population aged 16–64, ρ_s^{ess} is the state-specific essential-worker share, and ρ^{cond} is the national high-risk-condition share. Analogous formulas were used for other age thresholds.

The final eligibility measure was

$$E_{s,t} = \frac{N_{s,t}^{elig}}{N_s^{5+}}, \tag{2}$$

where N_s^{5+} is the state population aged 5 or older. The 5+ denominator was used because the eligibility series extends to the period when eligibility expanded to children aged 5 and above.

References

1. Ballotpedia. Coronavirus (COVID-19) vaccination rates and distribution plans by state. https://ballotpedia.org/Coronavirus_(COVID-19)_vaccination_rates_and_distribution_plans_by_state, 2021. Accessed 2025.

2. U.S. Census Bureau. Annual estimates of the civilian population by single year of age and sex for the united states and states: April 1, 2020 to july 1, 2024 (sc-est2024-agesex-civ). https://www.census.gov/data/tables/time-series/demo/popest/2020s-state-detail.html. U.S. Department of Commerce.

3. Larry Levitt, Samantha Artiga, Matthew Rae, Tricia Neuman, Gary Claxton, and Jennifer Kates. Estimates of the initial priority population for COVID-19 vaccination by state. https://www.kff.org/covid-19/estimates-of-the-initial-priority-population-for-covid-19-vaccination-by-state/, 2020. Kaiser Family Foundation Issue Brief.

4. United Way NCA. Us states with the most essential workers, 2020. URL https://unitedwaynca.org/blog/us-states-with-the-most-essential-workers/. Accessed December 25 2025.

5. Kathleen Dooling, Mona Marin, Megan Wallace, Nancy McClung, Mary Chamberland, Grace M. Lee, H. Keipp Talbot, José R. Romero, Beth P. Bell, and Sara E. Oliver. The Advisory Committee on Immunization Practices' updated interim recommendation for allocation of COVID-19 vaccine—United States, December 2020. *MMWR. Morbidity and Mortality Weekly Report*, 69(51-52):1657–1660, 2021. doi: 10.15585/mmwr.mm695152e2.
