# Supplementary material for: Population-level behavioral and structural drivers of COVID-19 vaccine uptake in the US
Source: PLoS Comput Biol. 2026 Jul 20;22(7):e1013988. doi: 10.1371/journal.pcbi.1013988 (PMC13405102; doi:10.1371/journal.pcbi.1013988)
Supplement: S3 File — (PDF) [file pcbi.1013988.s003.pdf]

Appendix C: Out-of-sample test results

This Appendix presents the state-level results of the out-of-sample validation exercise described in the main text. For each U.S. state and Washington, D.C., model parameters were estimated using the first 60% of the vaccination time series (weeks 0–46), and model performance was evaluated on the remaining weeks (47–77). Fig A3 compares the simulated vaccination trajectories against the observed data for all regions. For the out-of-sample proportion, across the regions, the average correlation between simulated and observed vaccination uptake is 0.55 with a standard deviation of 0.24.

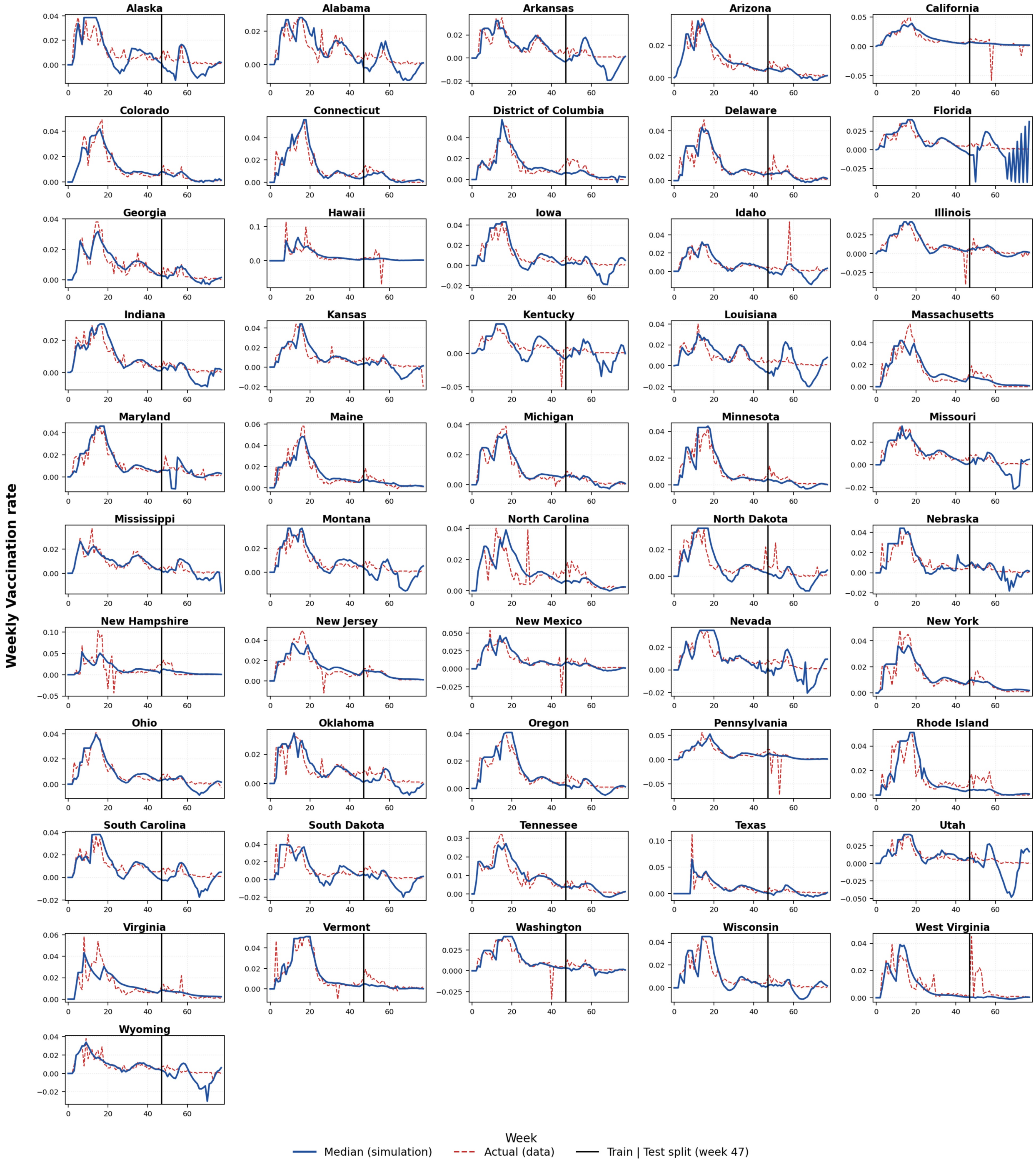

Fig A3: **Out-of-sample test** The solid blue line shows the median simulated weekly vaccination trajectory, while the red dashed line represents the observed data. The vertical black line at week 47 separates the training period (weeks 0–46), used for parameter estimation, from the out-of-sample test period (weeks 47–77).
