# Supplementary material for: Population-level behavioral and structural drivers of COVID-19 vaccine uptake in the US
Source: PLoS Comput Biol. 2026 Jul 20;22(7):e1013988. doi: 10.1371/journal.pcbi.1013988 (PMC13405102; doi:10.1371/journal.pcbi.1013988)
Supplement: Attachment — Submitted filename: author response.docx [file pcbi.1013988.s005.docx]

**PCOMPBIOL-D-26-00285**  
**Response to Reviewers**

Dear Editor,

We appreciate the opportunity to submit a revised version of our manuscript, entitled *Population-Level Behavioral and Structural Drivers of COVID-19 Vaccine Uptake in the US*, for consideration for publication in PLOS Computational Biology.

We have carefully considered all reviewer comments and revised the manuscript accordingly. In particular, we made the following major changes:

- We made substantial revisions throughout the manuscript, particularly in the Methods and Discussion sections. These changes provide a clearer explanation of the conceptual framework and further justify key modeling choices related to adoption dynamics and vaccination capacity.
- As requested by the reviewers, we conducted an out-of-sample validation analysis. The results are now presented in the main manuscript, with additional details provided in the Appendix.
- We added a new subsection in the Results section comparing our empirical findings on the mechanisms driving vaccination uptake over time. This analysis identifies three major phases of vaccine adoption. We also added a new figure in the main text, with additional supporting figures included in the Appendix.
- In response to reviewer requests, we added a new Appendix describing our reconstruction of state-level vaccine eligibility data. We also make these data available to readers for transparency and reproducibility.
- We added an Author Summary.

We provide a point-by-point response to all reviewer comments below, with our responses highlighted in blue. For ease of review, we reference the relevant manuscript sections where each revision has been made.

Thank you for your consideration. We look forward to hearing from you.

Sincerely,
The Authors

**Point-by-point response to reviewers’ comments**

(Reviewers’ comments start with **R#** and is in black. Authors responses start with **A#** and are in blue): Note: Pages in the paper are specified by **P**, and lines by **L**.

**Reviewer #1**

**R1-Summary:** This manuscript studies COVID-19 vaccine uptake in the United States, focusing on population-level behavioral and structural drivers through a parsimonious dynamic model based on an extended Bass diffusion framework. The topic is important, the paper is clearly motivated, and I think the focus on uptake dynamics rather than final coverage alone is a genuine strength. The attempt to combine a simple mechanistic model with empirical estimation across all 50 states and DC is also potentially valuable. At the same time, I think the manuscript would benefit from substantial revision before publication. My main concerns center on the modeling choices, the statistical specification, and reproducibility/documentation of key inputs. Overall, my recommendation is major revision.

**A1-Summary:** Thank you for the encouraging and constructive comments. We hope you find this revision satisfactory. Please see our responses below.

**R1-1:** Modeling choices need stronger justification and framing. The paper’s modeling strategy is interesting, but some core choices require clearer motivation. In particular, the use of an extended Bass diffusion model for vaccine uptake is not self-evident and deserves better exposition. Vaccination is not a standard product-adoption process, and here the framework is being used to represent a setting shaped by epidemic risk, eligibility rules, logistical rollout, and changing public perceptions. I think the authors should explain more explicitly why a Bass-style structure is appropriate in this context, what it captures especially well, and what its limitations are relative to more standard epidemic-behavior modeling approaches.

A1-1: We thank the reviewer for this comment. We agree that the use of a Bass diffusion framework in the context of vaccination requires clearer motivation and framing. In the revised manuscript, we have added a new paragraph (Section “Modeling”, page 4, in blue) that explicitly explains why a Bass-style structure is appropriate in this setting.

In particular, we clarify that our use of the Bass model is a parsimonious way to represent population-level feedback mechanisms. These include positive effects such as social influence and information diffusion, as well as negative effects such as free-riding, declining perceived risk, and selection bias when vaccine-enthusiasts vaccinate first. As such this model is not identical to the Bass diffusion model, but only inspired by it, and has more feasible modes of behavior in its structure. In fact, our empirical estimation does not find the ‘social influence’ channel to be prominent in this setting, at least in the laster stages of the COVID-19 vaccination hisotry.

We also emphasize that similar endogenous feedback structures arise in a wide range of epidemic–behavior models (e.g., Espinoza et al., 2024; Martcheva et al., 2021), even when not explicitly framed as diffusion processes. In many such models, the adoption rate (of a behavior, such as masking) depends on both the remaining non-adopter population and the current level of adoption, yielding dynamics that are closely related in structure to diffusion-type formulations.

We further note the connection to Epstein et al.’s “triple contagion” framework, where vaccination uptake is driven by behavioral responses to perceived risk. Our formulation is mathematically close to theirs but differs in that it provides a reduced-form specification that is directly amenable to empirical estimation in a regression framework across states, and we conduct the empirical estimation whereas their work is more theoretical.

We believe these revisions clarify both the rationale for our modeling choice and its scope.

Reference:

- Espinoza, B., Saad-Roy, C. M., Grenfell, B. T., Levin, S. A., & Marathe, M. V. (2024). Adaptive human behaviour modulates the impact of immune life history and vaccination on long-term epidemic dynamics. Proceedings of the Royal Society B: Biological Sciences, 291(2033), 20241772.
- Martcheva, M., Tuncer, N., & Ngonghala, C. N. (2021). Effects of social distancing on infectious disease dynamics: An evolutionary game theory and economic perspective. Journal of Biological Dynamics, 15(1), 342–366.
- Epstein, J. M., et al. (2021). Triple contagion: A two-fears epidemic model. Journal of The Royal Society Interface, 18(181), 20210186.

**R1-2:** Relatedly, the manuscript treats infection dynamics as exogenous and uses observed case data as a driver of vaccination behavior, while leaving a fully coupled epidemic-vaccination system for future work. That may be a reasonable simplification, but it should be framed more carefully. As written, the paper sometimes reads as if it is uncovering broader behavior-disease feedback mechanisms, whereas the actual model is better understood as a reduced-form uptake model conditional on observed epidemic conditions. I would encourage the authors to sharpen this framing and moderate the scope of their mechanistic claims accordingly.

A1-2: We agree that our framework is best interpreted as a model of risk response, here vaccination uptake, conditional on observed epidemic dynamics. In the revised manuscript, we have clarified this point by explicitly stating that infection cases are treated as exogenous inputs capturing the epidemiological environment faced by individuals (page 3, at the end of the introduction section).

We have also moderated the language to avoid suggesting that we model a fully coupled epidemic–behavior system (page 3). Our focus is on estimating behavioral responses in vaccination uptake given observed risk conditions. We believe this clarification better aligns the scope of the model with its intended interpretation.

**R1-3:** The capacity term is central but insufficiently justified. The treatment of vaccination capacity is an important aspect of the paper, yet it seems somewhat under-discussed. The model defines weekly capacity as the maximum observed vaccination increment up to time t, and this same quantity is then used to motivate the upper-censoring structure in the Tobit regression. This makes the capacity proxy look partly outcome-derived rather than independently measured. As a result, it is not entirely clear whether the model is separating true rollout constraints from realized demand, or whether these are being blended together. Because this proxy plays such a central role in both the dynamic model and the regression framework, I think the manuscript would benefit from a clearer justification of what this quantity represents, why it is a reasonable proxy for capacity, and how sensitive the results are to alternative formulations.

A1-3: Thank you for the comment. We have now added more explanations and justifications of the capacity measure in the methods section. We also acknowledge the inherent limitation of this outcome-derived measure (instead of independently measured) and discussed this in the limitation section. In addition, we also tested using the past 3 weeks’ rolling average of capacity constraint as an alternative formulation to smooth out the early single spike (now included in the robustness check section), and it did not meaningfully change our model fit.

Specifically, in the methods section we wrote:

“Weekly capacity denotes the maximum amount of vaccines each state can administer in a specific week, which is affected by various factors such as number of doses allocated and delivered, operational administration sites, staffing and scheduling. There is no reliable and consistent data at the state-week level for vaccination capacity. We therefore used an outcome-driven approach to estimate weekly capacity as the maximum observed increment up to time 𝑡:

This formulation reflects the intuition that the highest weekly uptake realized up to any point represents the ceiling of what the delivery system was able to process under the most favorable conditions observed so far. It is motivated by several features of the early U.S. COVID-19 vaccination rollout. First, vaccine supply and administration infrastructure expanded sharply over the first several months and the distribution network remained available once built (Santoli et al., 2024). Second, studies showed that early vaccination rates were consistently constrained by supply shipments, allocation quotas, and the number of available administration sites rather than by a lack of willing recipients (Hedge et al., 2023). Under these conditions, the historical maximum provides a natural, data-derived upper bound: weeks in which observed uptake fell well below this maximum are interpreted as capacity-unconstrained, while weeks at or near the maximum are treated as capacity-constrained.”

And in the discussion section we wrote:

“Finally, our proxy for capacity constraints is data-derived rather than independently measured and therefore cannot cleanly separate true delivery-system capacity from periods of peak demand. Our sensitivity analyses demonstrated that this capacity constraint proxy played a genuine structural role, as it was the main driver/constraint of vaccine uptake in the first 7-12 weeks, and removing it from the model resulted in meaningful deterioration of the model's performance. Future work could explore alternative formulations and assemble data to generate a more structurally grounded capacity measure.”

References

- Jeanne M. Santoli, Cameron Noblit Lori B. Moore Roua El Kalach Duggar, Christopher and Carolyn B. Bridges. US covid-19 vaccine distribution strategies, systems, performance, and lessons learned, December 2020–May 2023. Vaccine, 42(125703), 2024. doi: 10.1016/j.vaccine.2024.02.020.
- S. J. Hegde, H. Mahmassani, and K. Smilowitz. A two-regime analysis of the covid-19 vaccine distribution process in the united states. Journal of Humanitarian Logistics and Supply Chain Management, 13(2):290–312, 2023. doi: 10.1108/JHLSCM-10-2021-0106.

**R1-4:** The Tobit specification and what is actually identified need fuller discussion. The ODE-informed Tobit regression is an interesting feature of the manuscript, but I think the statistical reasoning behind it could be explained more fully. In particular, it would help to clarify why Tobit is the most appropriate estimator for this setting, what assumptions are required for its interpretation, and what exactly the estimated coefficients should be understood to identify in this framework. The discussion could also more explicitly address how the exclusion of negative weekly increments, attributed to reporting corrections, may affect estimation and interpretation.

A1-4: Thank you. In the methods we have now explained more on the choice of Tobit model, the inherent assumptions and the implications of excluding negative weekly increments. Specifically we wrote:

“The upper-censored Tobit model is appropriate here because the dependent variable 𝑦𝑡 is structurally censored from above by the capacity ratio 𝐶𝑡 /(𝐸𝑡−1 − 𝑉𝑡−1): when capacity constraint is binding, observed uptake equals the capacity ceiling regardless of the underlying behavioral propensity, so OLS would systematically underestimate the underlying parameters. The Tobit likelihood corrects for this by treating censored and uncensored weeks differently: uncensored weeks contribute through estimating the exact density function in the absence of the ceiling, whereas censored weeks contribute through estimating the probability of exceeding the ceiling. While both observations contribute information to the estimation process, the behavioral parameters 𝛽0, 𝛽1, and 𝛽2 are identified heavily from weeks in which the capacity constraint was not binding and thus more closely reflect the underlying (uncensored) parameters (Amemiya, 1984). Consistent estimation assumes the censoring threshold is correctly specified, errors are normally distributed, and the latent model (uncensored) is linear. During estimation we only used observations where 𝑑𝑉𝑡 ≥ 0, as negative 𝑑𝑉𝑡 can emerge due to reporting errors and corrections that do not reflect the true behavioral mechanism. We note that while this choice should result in more reliable estimates of the behavioral parameters, it could also result in a worse predictive fit of the cumulative vaccination 𝑉𝑡 if there are frequent or large corrections.”

Reference:

- Amemiya, T. (1984). Tobit models: A survey. *Journal of econometrics*, *24*(1-2), 3-61.

**R1-5:** More generally, the current model evaluation appears to rely largely on in-sample fit: coefficients are estimated from each state’s observed time series and the resulting simulations are then compared to those same trajectories. This is certainly informative as a descriptive exercise, but it may be less informative about predictive validity or the stability of the inferred mechanisms. For that reason, I think the paper would be strengthened by including at least one out-of-sample validation exercise, such as holding out the last part of each state’s series, using rolling-origin forecasts, or estimating on one phase of the rollout and testing on another. Comparisons against simpler benchmark models would also help clarify how much explanatory value is added by the proposed framework.

A1-5: We thank the reviewer for this valuable suggestion. In response, we have added an out-of-sample validation exercise in the revised manuscript. Specifically, for each state (including Washington, DC), model parameters were estimated using only the first 60% of the time series (weeks 0--46). The estimated parameters were then held fixed, and the model was simulated over the rest of the time horizon (weeks 47--77).

In short, across the 50 states and Washington, DC, the average correlation between simulated and observed vaccination uptake in the hold-out period was 0.55 (SD = 0.24). For comparison, when the same time period was included in the calibration window (i.e., evaluated in-sample), the corresponding average correlation was 0.65 (SD = 0.21). This relatively small decline in correlation suggests that the model captures behavioral uptake dynamics that generalize beyond the estimation window.

These results are now reported in the manuscript and provide additional evidence that the model captures vaccine uptake dynamics beyond in-sample fitting. Specifically, a new paragraph is added to the method section; a sub-section is added to the results section, and detailed state-level figures are provided in the new Appendix C.

**R1-6:** Documentation of the eligibility reconstruction. I found the reconstruction of state-level vaccine eligibility over time to be potentially very valuable, and I think the manuscript could highlight this contribution more clearly. Since eligibility enters directly into the model through the E_t term, readers would likely benefit from a somewhat fuller description of how the state-level timelines were reconstructed from the underlying policy documents and translated into the EtE_tEt series used in the analysis. A brief appendix or supplementary note outlining the coding procedure, and if feasible sharing the resulting state-by-week eligibility coding in a reusable form, would make this part of the study easier to follow and would further strengthen its reproducibility.

A1-6: We thank the reviewer for this helpful suggestion. We agree, and we have added a new Supplementary Appendix B: Reconstruction of state-level vaccine eligibility describing the coding procedure end-to-end, and we now release the full state-by-week coding as a reusable supplementary dataset.

**R1-7:** Presentation of the main findings could be improved. A major aim of the paper is to uncover the mechanisms behind vaccination uptake over time. For that reason, I found it somewhat strange that the mechanism decomposition is shifted to the Appendix, even though it directly supports one of the manuscript’s central claims: namely, that the early phase of rollout was driven mainly by eligibility and capacity constraints, while later dynamics were more behaviorally driven and responsive to risk. I think the main text would benefit from bringing at least part of that decomposition analysis forward and discussing it more fully.

A1-7: We thank the Reviewer for this constructive suggestion, which substantially strengthens the paper. We have restructured the manuscript so that the “mechanism decomposition” now is in the main text. A new Results subsection, "Comparing the mechanisms," presents the analysis together with a five-panel figure (Figure 6) that combines: the cross-state median trajectory of each mechanism with a 50 percent range across the 51 U.S. jurisdictions (Panel A); a quantitative summary of how the relative roles of the four mechanisms shift over time, expressed as correlations between observed weekly vaccination and each mechanism within five 15-week intervals (Panel B); and the deterministic decomposition for three illustrative states,

**R1-8:** Conversely, the full state-by-state prediction panels are informative but visually dense. I would suggest showing a smaller set of representative states in the main text, including both good and poor fits, and moving the complete multi-panel state figures to the appendix or supplement. I offer this as a suggestion for readability rather than a strict condition.

A1-8: We thank the reviewer for this suggestion and agree that the full state-level panels are visually dense. After considering this recommendation, we decided to retain the figure in the main text, as we believe it provides an important overview of model performance across a large and heterogeneous set of regions. Moving it to the Appendix could reduce its visibility, as appendix material is often consulted less frequently, and we already have a few more figures in this revision in the Appendix. In particular, this figure allows readers to directly assess both the extent of data used for parameter estimation and how the simulated trajectories compare with observed vaccination uptake across states. To make the figure more readable we modified the figure to have 5 columns, and increases its font size, and we think this slightly improved it.

**R1-9:** In the Figure 1 caption, “Republication” should be corrected to “Republican.”

A1-9: Thank you. We have now fixed this error.

**R1-10:** The final section title reads “Discussions”; “Discussion” would be more standard.

A1-10: Thank you. We have now fixed this.

**Reviewer #2**

**R2-Summary:** This paper introduces a framework for studying the dynamical aspects of vaccination rates. The authors apply their model to COVID-19 vaccinations in the US from 2020-2022. The developed framework enables an estimate of different parameters associated with each US state, the parameters describing the proportional adoption, risk driven adoption, and vaccination-dependent adoption. The authors find two distinct phases in the vaccination rates: the first best fit by proportional and vaccination-dependent adoption, the second by risk driven adoption. The authors end by discussing possible extensions to their framework.

Strengths: This work studies an important question - how does vaccination rates change with time and what drives those dynamics? The authors provide a simple, but effective framework and use it to derive insight into: 1) differences between states; 2) differences between possible drivers; 3) differences across the COVID-19 pandemic. Figure 2 was helpful in clearly explaining the authors' framework. The Introduction was well motivated and the Discussion identified a number of important future directions for extending this work.

**A2-Summary:** Thank you for the encouraging comment on the strengths. Please see our responses to the rest of your comments below.

**R2-1:** The authors did not ever (as far as I could tell) define exactly what they did when using the Tobit regression model. No references were cited and the actual implementation was not described. I think for increasing clarity, these details are necessary.

**A2-1:** Thank you. In response to the comment and a comment from reviewer 1, in the Methods we have now explained more on the choice of Tobit model, the inherent assumptions. Specifically, we wrote:

“The upper-censored Tobit model is appropriate here because the dependent variable 𝑦𝑡 is structurally censored from above by the capacity ratio 𝐶𝑡 /(𝐸𝑡−1 − 𝑉𝑡−1): when capacity constraint is binding, observed uptake equals the capacity ceiling regardless of the underlying behavioral propensity, so OLS would systematically underestimate the underlying parameters. The Tobit likelihood corrects for this by treating censored and uncensored weeks differently: uncensored weeks contribute through estimating the exact density function in the absence of the ceiling, whereas censored weeks contribute through estimating the probability of exceeding the ceiling. While both observations contribute information to the estimation process, the behavioral parameters 𝛽0, 𝛽1, and 𝛽2 are identified heavily from weeks in which the capacity constraint was not binding and thus more closely reflect the underlying (uncensored) parameters (Amemiya, 1984). Consistent estimation assumes the censoring threshold is correctly specified, errors are normally distributed, and the latent model (uncensored) is linear. During estimation we only used observations where 𝑑𝑉𝑡 ≥ 0, as negative 𝑑𝑉𝑡 can emerge due to reporting errors and corrections that do not reflect the true behavioral mechanism. We note that while this choice should result in more reliable estimates of the behavioral parameters, it could also result in a worse predictive fit of the cumulative vaccination 𝑉𝑡 if there are frequent or large corrections.”

Reference:

- Amemiya, T. (1984). Tobit models: A survey. *Journal of econometrics*, *24*(1-2), 3-61.

**R2-2:** To me, one of the most interesting results the authors find is that the later vaccination rates are most strongly driven by risk adoption. However, the evidence for this claim is not presented in the main text. In addition, the figure that demonstrates this (Fig. A.1) is in the Appendix and does not provide much additional detail. I think: 1) this figure should be moved to the main text; 2) more states (beyond just the CA, NY, TN) should be included; 3) more detail be provided on what exactly each of the curves is showing (I assume each one is using only one of the 3 variables in the model); 4) a summary plot showing the average error in fit vaccination rates, using each of the 3 models, for all states should be included (this would show the point most strongly, that there is a transition).

A2-2: We thank the Reviewer for these constructive recommendations, which we have largely incorporated by adding a new sub-section to Results section of the paper (Comparing the mechanisms) and extending Appendix A. Here we review each of the four points listed above:

1) We agree. It now appears as Figure 6 in a new Results subsection, "Comparing the mechanisms," with an expanded discussion that draws out the implications the Reviewer raised in the original review.

2) To broaden the state coverage without overwhelming the main-text figure, we now follow this approach: The main-text figure retains California, New York, and Tennessee as illustrative cases (Panels C–E), and adds a new Panel A that summarizes the typical behavior across all 51 U.S. jurisdictions using the cross-state median with a shaded 50 percent range. The full set of state-level decompositions for every state and DC is now provided in Appendix A, in a 5×11 small-multiples figure (Figure A.2) so that any individual jurisdiction can be inspected directly.

3) The figure caption and accompanying text in the new sub-section now describe each element of the decomposition explicitly. Please see the new sub-section.

4) We considered this suggestion carefully. The exact procedure the Reviewer describes would require fitting a separate single-mechanism reduced model for each behavioral term and reporting the time-resolved fit error of each. This needs independent re-estimation of all model coefficients for each reduced specification, since in our paper the coefficients are jointly identified within the full model. In order to at least partially address this point, we conducted a correlational analysis and now offer a new Panel in Figure 6, Panel B. This panel reports the correlation between observed weekly vaccination and each of the four mechanisms within five 15-week intervals, computed across all (state, week) observations in each interval. Taken together with the magnitude evidence in Panel A and the point-to-point correlation offered in Panel B, we can better see that a) supply constraints bind in the early phase while b) proportional adoption generates the underlying demand, c) risk-responsive behavior governs mid-phase fluctuations once the capacity ceiling relaxes, as well as point-to-point correlations, and d) saturation dynamics dominate the late phase.

**R2-3:** The authors mention that there is a systematic bias in the cumulative vaccination rates of their fit model (Fig. 5), but it was not clear to me why this was. This seems to be an important feature, so more discussion on this would be clarifying.

A2-3: Thank you. We have now further clarified this point. Specifically we wrote:

“There is a minor discrepancy between the simulations and data in many states: right after the main peak in many states vaccination rates drop faster than what our models predicts. As a result the observed total vaccination coverage stayed slightly lower than the model predictions since after that peak. This may partially be due to rapid vaccination among those most interested in vaccines, which leaves a more reluctant pool of unvaccinated. While our model accounts for this effect through a linear effect of vaccinated-dependent uptake, the actual effect is likely nonlinear and we would need more complex specifications to improve the fit.”

**R2-4:** Fig. 1, while illustrative of the authors claim that vaccination rate dynamics differ across states, is confusing in two respects. First, Fig. 1b, which describes "weekly vaccinations" has negative values. I assume this is because what is being plotted is the derivative of weekly vaccinations, but this is not ever explicitly mentioned. And second, Fig. 1a, which describes the "cumulative vaccination", experiences drops. Why this is (when this is a cumulative plot) is not clear. Is this due to data reporting errors?

A2-4: Thank you for noticing this. Fig.1a plots V over time and Fig.1b plots dV over time. The drop in V and negative value in dV are due to reporting errors and corrections in the raw data. We have now explained this in the figure caption.

**R2-5:** (very minor) "Second, We incorporate vaccination capacity, which has been shown to limit the vaccination rate, particularly during the early rollout [20]. Third, We included age-specific vaccination eligibility, reflecting the phased expansion of vaccine eligibility from older and high- risk populations to younger, general populations [19]." has multiple capitalizations.

A2-5: Thank you. We have now fixed this.

**R2-6:** (very minor) The authors switch between referring to states by their name and the abbreviation.

A2-6: Thank you. We have now referred to each state by full name in the main text.

**R2-7:** (very minor) In the Results section, the writing switches to including a lot of sentences with colons. This is perfectly fine, but feels a little out of character given the writing in the other sections.

A2-7: Thank you. We have now removed some colons and made the writing style consistent with other sections.

**R2-8:** Overall thoughts: I enjoyed this paper and I think it tackles an important and interesting question about vaccination DYNAMICS. It seems suited for PLOS Comp Bio. My main reservations are in details relating to: 1) the model (Tobit); 2) more detailed presentation of a major claim (later phase of vaccination driven by risk); 3) data presented in Fig. 1 (which is the data studied by the authors in the paper). Addressing all of these should be straightforward, but I think necessary for acceptance.

A2-8: Thank you for your careful review and constructive comments. We hope the new revision addresses your comments.
