# Supplementary material for: Population-level behavioral and structural drivers of COVID-19 vaccine uptake in the US
Source: PLoS Comput Biol. 2026 Jul 20;22(7):e1013988. doi: 10.1371/journal.pcbi.1013988 (PMC13405102; doi:10.1371/journal.pcbi.1013988)
Supplement: Attachment — Submitted filename: author_response_auresp_2.docx [file pcbi.1013988.s006.docx]

**PCOMPBIOL-D-26-00285**  
**Response to Reviewers**

Dear Editor,

We appreciate the minor revision decision of our manuscript, entitled *Population-Level Behavioral and Structural Drivers of COVID-19 Vaccine Uptake in the US*, for consideration for publication in PLOS Computational Biology.

We have carefully considered all reviewer comments and revised the manuscript accordingly. We provide a point-by-point response to all reviewer comments below, with our responses highlighted in blue.

Thank you for your consideration. We look forward to hearing from you.

Sincerely,
The Authors

**Point-by-point response to reviewers’ comments**

(Reviewers’ comments start with **R#** and is in black. Authors responses start with **A#** and are in blue): Note: Pages in the paper are specified by **P**, and lines by **L**.

**Reviewer #1**

**R1:** I think the authors have quite satisfactorily amended the major issues flagged in the first round of revision. I sinsceresly congratulate them as I find now the manuscript ready for publication.

**A1:** Thank you!

**Reviewer #2**

**R2-Summary:** I thank the authors for the detailed revisions which greatly improve the quality of the manuscript and clarify a number of points that I had questions on. In particular, I think the added text around the Tolbit regression was very helpful and the new analysis of the 3 phases of vaccine uptake strengthened the point. In addition, the role of corrections to reported vaccine data in Fig. 1 was clarifying. I have two points (both straightforward) that I think need to be addressed in the final version of the paper:

**A2-Summary:** Thank you. Please see our responses to the rest of your comments below.

**R2-1:** I appreciate the authors presenting the decomposition of all 51 states and regions in Fig. A.2. However, it appears that the true weekly vaccinations were never added to the plots. In all 51 plots, there is a solid black line a 0, which would suggest that there was no weekly vaccination for any of the states.

**A2-1:** Thank you for catching that. We have now added weekly vaccinations in Fig. A.2.

**R2-2:** The author summary starts with "Why do some communities vaccinate quickly while others lag behind—even when vaccines are equally available?" I think this is a very interesting and catchy question, and certainly the authors method (through the decomposition) could address this. However, besides just plotting the individual decomposition curves in Fig. 6C-E and Fig. A.2, the authors do not really address what is different between the different states. I think therefore the authors should either change their motivating question to reflect the results they investigate in detail (e.g., the 3 different phases) or they should add more analysis to what differs between the different states (e.g., the authors compute the correlations of each component of the decomposition for all states with time).

**A2-2**: Good point. We now revised the author summary as:

“How does COVID-19 vaccination uptake change over time, and what forces drive the vaccination uptake? We set out to understand what mechanisms shaped the COVID-19 vaccination rates and how these mechanisms evolved across the 2021–2022 rollout in the United States. We built a mathematical model that treats vaccination as a behavior that evolves in response to how many people are already vaccinated, how severe the local outbreak appears, and whether vaccines are accessible. Using weekly vaccination records from all 50 states and Washington, D.C., we estimated the strength of each force for every state. We found that our simple model can reproduce the observed vaccination trajectories, and vaccination unfolded in three phases: an early period when limited supplies constrained uptake, a middle period when rising case numbers prompted more people to get vaccinated, and a later period when uptake slowed as the most willing recipients had already been reached. States also differed substantially in how strongly residents responded to infection risk and in their overall tendency to seek vaccination when doses were available.”

In the limitation paragraph in the discussion section we also added:

“Third, our model specification is necessarily aggregate and does not explicitly capture within-state heterogeneity by age, socioeconomic status, or political affiliation, all of which are known to influence vaccination decisions [47, 48, 17]. Similarly, while we document substantial cross-state heterogeneity in behavioral parameters such as baseline willingness to vaccinate and responsiveness to infection risk, we do not systematically link these differences to observable state characteristics. Future studies could incorporate these factors, examine the correlates of estimated behavioral parameters across states, and build models on a more detailed level.”
