# Supplementary material for: Population-level behavioral and structural drivers of COVID-19 vaccine uptake in the US
Source: PLoS Comput Biol. 2026 Jul 20;22(7):e1013988. doi: 10.1371/journal.pcbi.1013988 (PMC13405102; doi:10.1371/journal.pcbi.1013988)
Supplement: Peer review history

# Peer review: Decision Letter 0

**DOI:** 10.1371/journal.pcbi.1013988.r001

**Authors:** Yamir Moreno, Jennifer Flegg

27 Apr 2026

PCOMPBIOL-D-26-00285

Population-Level Behavioral and Structural Drivers of COVID-19 Vaccine Uptake in the US

PLOS Computational Biology

Dear Dr. Xu,

Thank you for submitting your manuscript to PLOS Computational Biology. After careful consideration, we feel that it has merit but does not fully meet PLOS Computational Biology's publication criteria as it currently stands. Therefore, we invite you to submit a revised version of the manuscript that addresses the points raised during the review process through a major revision.

Please submit your revised manuscript by Jun 27 2026 11:59PM. If you will need more time than this to complete your revisions, please reply to this message or contact the journal office at ploscompbiol@plos.org. When you're ready to submit your revision, log on to https://www.editorialmanager.com/pcompbiol/ and select the 'Submissions Needing Revision' folder to locate your manuscript file.

Please include the following items when submitting your revised manuscript:

* A letter that responds to each point raised by the editor and reviewer(s). You should upload this letter as a separate file labeled 'Response to Reviewers'. This file does not need to include responses to formatting updates and technical items listed in the 'Journal Requirements' section below.

* A marked-up copy of your manuscript that highlights changes made to the original version. You should upload this as a separate file labeled 'Revised Manuscript with Track Changes'.

* An unmarked version of your revised paper without tracked changes. You should upload this as a separate file labeled 'Manuscript'.

If you would like to make changes to your financial disclosure, competing interests statement, or data availability statement, please make these updates within the submission form at the time of resubmission. Guidelines for resubmitting your figure files are available below the reviewer comments at the end of this letter

We look forward to receiving your revised manuscript.

Kind regards,

Yamir Moreno

Academic Editor

PLOS Computational Biology

Jennifer Flegg

Section Editor

PLOS Computational Biology

**Journal Requirements:**

1) We ask that a manuscript source file is provided at Revision. Please upload your manuscript file as a .doc, .docx, .rtf or .tex. If you are providing a .tex file, please upload it under the item type u2018LaTeX Source Fileu2019 and leave your .pdf version as the item type u2018Manuscriptu2019.

2) Please provide an Author Summary. This should appear in your manuscript between the Abstract (if applicable) and the Introduction, and should be 150-200 words long. The aim should be to make your findings accessible to a wide audience that includes both scientists and non-scientists. Sample summaries can be found on our website under Submission Guidelines:

https://journals.plos.org/ploscompbiol/s/submission-guidelines#loc-parts-of-a-submission

3) Please upload all main figures as separate Figure files in .tif or .eps format. For more information about how to convert and format your figure files please see our guidelines:

https://journals.plos.org/ploscompbiol/s/figures

4) We notice that your supplementary Figures are included in the manuscript file. Please remove them and upload them with the file type 'Supporting Information'. Please ensure that each Supporting Information file has a legend listed in the manuscript after the references list.

5) Please amend your detailed Financial Disclosure statement. This is published with the article. It must therefore be completed in full sentences and contain the exact wording you wish to be published.

1) State the initials, alongside each funding source, of each author to receive each grant. For example: "This work was supported by the National Institutes of Health (####### to AM; ###### to CJ) and the National Science Foundation (###### to AM)."

2) State what role the funders took in the study. If the funders had no role in your study, please state: "The funders had no role in study design, data collection and analysis, decision to publish, or preparation of the manuscript."

6) Thank you for stating that "Data and code to replicate this study can be found below. https://www.dropbox.com/scl/fo/y3e7zz2xut4g5nufi5c0k/AI0ZahV7ago0htB4VrSMJEU?rlkey=fc6ai2jkpy2n4x967eox7ybtw&st=5z7uj369&dl=0."

Unfortunately, the repository you have noted in your Data Availability statement does not qualify as an acceptable data repository according to PLOS's standards.

At this time, please upload the minimal data set necessary to replicate your study's findings to a stable, public repository (such as figshare or Dryad) and provide us with the relevant URLs, DOIs, or accession numbers that may be used to access these data. For a list of recommended repositories and additional information on PLOS standards for data deposition, please see

https://journals.plos.org/ploscompbiol/s/recommended-repositories

Note: If the reviewer comments include a recommendation to cite specific previously published works, please review and evaluate these publications to determine whether they are relevant and should be cited. There is no requirement to cite these works unless the editor has indicated otherwise.

**Reviewers' comments:**

Reviewer's Responses to Questions

Reviewer #1: This manuscript studies COVID-19 vaccine uptake in the United States, focusing on population-level behavioral and structural drivers through a parsimonious dynamic model based on an extended Bass diffusion framework. The topic is important, the paper is clearly motivated, and I think the focus on uptake dynamics rather than final coverage alone is a genuine strength. The attempt to combine a simple mechanistic model with empirical estimation across all 50 states and DC is also potentially valuable. At the same time, I think the manuscript would benefit from substantial revision before publication. My main concerns center on the modeling choices, the statistical specification, and reproducibility/documentation of key inputs. Overall, my recommendation is major revision.

### Major comments

#### 1. Modeling choices need stronger justification and framing

The paper’s modeling strategy is interesting, but some core choices require clearer motivation. In particular, the use of an extended Bass diffusion model for vaccine uptake is not self-evident and deserves better exposition. Vaccination is not a standard product-adoption process, and here the framework is being used to represent a setting shaped by epidemic risk, eligibility rules, logistical rollout, and changing public perceptions. I think the authors should explain more explicitly why a Bass-style structure is appropriate in this context, what it captures especially well, and what its limitations are relative to more standard epidemic-behavior modeling approaches.

Relatedly, the manuscript treats infection dynamics as exogenous and uses observed case data as a driver of vaccination behavior, while leaving a fully coupled epidemic-vaccination system for future work. That may be a reasonable simplification, but it should be framed more carefully. As written, the paper sometimes reads as if it is uncovering broader behavior-disease feedback mechanisms, whereas the actual model is better understood as a reduced-form uptake model conditional on observed epidemic conditions. I would encourage the authors to sharpen this framing and moderate the scope of their mechanistic claims accordingly.

#### 2. The capacity term is central but insufficiently justified

The treatment of vaccination capacity is an important aspect of the paper, yet it seems somewhat under-discussed. The model defines weekly capacity as the maximum observed vaccination increment up to time t, and this same quantity is then used to motivate the upper-censoring structure in the Tobit regression. This makes the capacity proxy look partly outcome-derived rather than independently measured. As a result, it is not entirely clear whether the model is separating true rollout constraints from realized demand, or whether these are being blended together.

Because this proxy plays such a central role in both the dynamic model and the regression framework, I think the manuscript would benefit from a clearer justification of what this quantity represents, why it is a reasonable proxy for capacity, and how sensitive the results are to alternative formulations.

#### 3. The Tobit specification and what is actually identified need fuller discussion

The ODE-informed Tobit regression is an interesting feature of the manuscript, but I think the statistical reasoning behind it could be explained more fully. In particular, it would help to clarify why Tobit is the most appropriate estimator for this setting, what assumptions are required for its interpretation, and what exactly the estimated coefficients should be understood to identify in this framework. The discussion could also more explicitly address how the exclusion of negative weekly increments, attributed to reporting corrections, may affect estimation and interpretation.

More generally, the current model evaluation appears to rely largely on in-sample fit: coefficients are estimated from each state’s observed time series and the resulting simulations are then compared to those same trajectories. This is certainly informative as a descriptive exercise, but it may be less informative about predictive validity or the stability of the inferred mechanisms. For that reason, I think the paper would be strengthened by including at least one out-of-sample validation exercise, such as holding out the last part of each state’s series, using rolling-origin forecasts, or estimating on one phase of the rollout and testing on another. Comparisons against simpler benchmark models would also help clarify how much explanatory value is added by the proposed framework.

#### 4. Documentation of the eligibility reconstruction

I found the reconstruction of state-level vaccine eligibility over time to be potentially very valuable, and I think the manuscript could highlight this contribution more clearly. Since eligibility enters directly into the model through the E_t term, readers would likely benefit from a somewhat fuller description of how the state-level timelines were reconstructed from the underlying policy documents and translated into the EtE_tEt series used in the analysis. A brief appendix or supplementary note outlining the coding procedure, and if feasible sharing the resulting state-by-week eligibility coding in a reusable form, would make this part of the study easier to follow and would further strengthen its reproducibility.

#### 5. Presentation of the main findings could be improved

A major aim of the paper is to uncover the mechanisms behind vaccination uptake over time. For that reason, I found it somewhat strange that the mechanism decomposition is shifted to the Appendix, even though it directly supports one of the manuscript’s central claims: namely, that the early phase of rollout was driven mainly by eligibility and capacity constraints, while later dynamics were more behaviorally driven and responsive to risk. I think the main text would benefit from bringing at least part of that decomposition analysis forward and discussing it more fully.

Conversely, the full state-by-state prediction panels are informative but visually dense. I would suggest showing a smaller set of representative states in the main text, including both good and poor fits, and moving the complete multi-panel state figures to the appendix or supplement. I offer this as a suggestion for readability rather than a strict condition.

### Minor comments

Just a couple of typos:

- In the Figure 1 caption, “Republication” should be corrected to “Republican.”

- The final section title reads “Discussions”; “Discussion” would be more standard.

Reviewer #2: Summary: This paper introduces a framework for studying the dynamical aspects of vaccination rates. The authors apply their model to COVID-19 vaccinations in the US from 2020-2022. The developed framework enables an estimate of different parameters associated with each US state, the parameters describing the proportional adoption, risk driven adoption, and vaccination-dependent adoption. The authors find two distinct phases in the vaccination rates: the first best fit by proportional and vaccination-dependent adoption, the second by risk driven adoption. The authors end by discussing possible extensions to their framework.

Strengths: This work studies an important question - how does vaccination rates change with time and what drives those dynamics? The authors provide a simple, but effective framework and use it to derive insight into: 1) differences between states; 2) differences between possible drivers; 3) differences across the COVID-19 pandemic. Figure 2 was helpful in clearly explaining the authors' framework. The Introduction was well motivated and the Discussion identified a number of important future directions for extending this work.

Weaknesses:

1. The authors did not ever (as far as I could tell) define exactly what they did when using the Tobit regression model. No references were cited and the actual implementation was not described. I think for increasing clarity, these details are necessary.

2. To me, one of the most interesting results the authors find is that the later vaccination rates are most strongly driven by risk adoption. However, the evidence for this claim is not presented in the main text. In addition, the figure that demonstrates this (Fig. A.1) is in the Appendix and does not provide much additional detail. I think: 1) this figure should be moved to the main text; 2) more states (beyond just the CA, NY, TN) should be included; 3) more detail be provided on what exactly each of the curves is showing (I assume each one is using only one of the 3 variables in the model); 4) a summary plot showing the average error in fit vaccination rates, using each of the 3 models, for all states should be included (this would show the point most strongly, that there is a transition).

3. The authors mention that there is a systematic bias in the cumulative vaccination rates of their fit model (Fig. 5), but it was not clear to me why this was. This seems to be an important feature, so more discussion on this would be clarifying.

4. Fig. 1, while illustrative of the authors claim that vaccination rate dynamics differ across states, is confusing in two respects. First, Fig. 1b, which describes "weekly vaccinations" has negative values. I assume this is because what is being plotted is the derivative of weekly vaccinations, but this is not ever explicitly mentioned. And second, Fig. 1a, which describes the "cumulative vaccination", experiences drops. Why this is (when this is a cumulative plot) is not clear. Is this due to data reporting errors?

Minor points:

1. (very minor) "Second, We incorporate vaccination capacity, which has been shown to limit the vaccination rate, particularly during the early rollout [20]. Third, We included age-specific vaccination eligibility, reflecting the phased expansion of vaccine eligibility from older and high- risk populations to younger, general populations [19]." has multiple capitalizations.

2. (very minor) The authors switch between referring to states by their name and the abbreviation.

3. (very minor) In the Results section, the writing switches to including a lot of sentences with colons. This is perfectly fine, but feels a little out of character given the writing in the other sections.

Overall thoughts: I enjoyed this paper and I think it tackles an important and interesting question about vaccination DYNAMICS. It seems suited for PLOS Comp Bio. My main reservations are in details relating to: 1) the model (Tobit); 2) more detailed presentation of a major claim (later phase of vaccination driven by risk); 3) data presented in Fig. 1 (which is the data studied by the authors in the paper). Addressing all of these should be straightforward, but I think necessary for acceptance.

**********

**Have the authors made all data and (if applicable) computational code underlying the findings in their manuscript fully available?**

The PLOS Data policy requires authors to make all data and code underlying the findings described in their manuscript fully available without restriction, with rare exception (please refer to the Data Availability Statement in the manuscript PDF file). The data and code should be provided as part of the manuscript or its supporting information, or deposited to a public repository. For example, in addition to summary statistics, the data points behind means, medians and variance measures should be available. If there are restrictions on publicly sharing data or code —e.g. participant privacy or use of data from a third party—those must be specified.

Reviewer #1: Yes

Reviewer #2: Yes

**********

PLOS authors have the option to publish the peer review history of their article (what does this mean?). If published, this will include your full peer review and any attached files.

If you choose “no”, your identity will remain anonymous but your review may still be made public.

**Do you want your identity to be public for this peer review?**  For information about this choice, including consent withdrawal, please see our Privacy Policy.

Reviewer #1: No

Reviewer #2: **Yes:** William Redman

[NOTE: If reviewer comments were submitted as an attachment file, they will be attached to this email and accessible via the submission site. Please log into your account, locate the manuscript record, and check for the action link "View Attachments". If this link does not appear, there are no attachment files.]

**Figure resubmission:**

While revising your submission, we strongly recommend that you use PLOS’s NAAS tool (https://ngplosjournals.pagemajik.ai/artanalysis) to test your figure files. NAAS can convert your figure files to the TIFF file type and meet basic requirements (such as print size, resolution), or provide you with a report on issues that do not meet our requirements and that NAAS cannot fix.

After uploading your figures to PLOS’s NAAS tool - https://ngplosjournals.pagemajik.ai/artanalysis, NAAS will process the files provided and display the results in the "Uploaded Files" section of the page as the processing is complete. If the uploaded figures meet our requirements (or NAAS is able to fix the files to meet our requirements), the figure will be marked as "fixed" above. If NAAS is unable to fix the files, a red "failed" label will appear above. When NAAS has confirmed that the figure files meet our requirements, please download the file via the download option, and include these NAAS processed figure files when submitting your revised manuscript.

**Reproducibility:**

To enhance the reproducibility of your results, we recommend that authors of applicable studies deposit laboratory protocols in protocols.io, where a protocol can be assigned its own identifier (DOI) such that it can be cited independently in the future. Additionally, PLOS ONE offers an option to publish peer-reviewed clinical study protocols. Read more information on sharing protocols at https://plos.org/protocols?utm_medium=editorial-email&utm_source=authorletters&utm_campaign=protocols

# Author comment: Author response to Decision Letter 1

**DOI:** 10.1371/journal.pcbi.1013988.r002

22 May 2026

Attachment Submitted filename: author response.docx

# Peer review: Decision Letter 1

**DOI:** 10.1371/journal.pcbi.1013988.r003

**Authors:** Yamir Moreno, Jennifer Flegg

9 Jun 2026

PCOMPBIOL-D-26-00285R1

Population-Level Behavioral and Structural Drivers of COVID-19 Vaccine Uptake in the US

PLOS Computational Biology

Dear Dr. Xu,

Thank you for submitting your manuscript to PLOS Computational Biology. After careful consideration, we feel that it will likely be accepted for publication if a few remaining minor points are successfully addressed. Therefore, we invite you to submit a revised version of the manuscript.

Please submit your revised manuscript by Aug 09 2026 11:59PM. If you will need more time than this to complete your revisions, please reply to this message or contact the journal office at ploscompbiol@plos.org. When you're ready to submit your revision, log on to https://www.editorialmanager.com/pcompbiol/ and select the 'Submissions Needing Revision' folder to locate your manuscript file.

Please include the following items when submitting your revised manuscript:

* A letter that responds to each point raised by the editor and reviewer(s). You should upload this letter as a separate file labeled 'Response to Reviewers'. This file does not need to include responses to formatting updates and technical items listed in the 'Journal Requirements' section below.

* A marked-up copy of your manuscript that highlights changes made to the original version. You should upload this as a separate file labeled 'Revised Manuscript with Track Changes'.

* An unmarked version of your revised paper without tracked changes. You should upload this as a separate file labeled 'Manuscript'.

If you would like to make changes to your financial disclosure, competing interests statement, or data availability statement, please make these updates within the submission form at the time of resubmission. Guidelines for resubmitting your figure files are available below the reviewer comments at the end of this letter.

As the corresponding author, your ORCID iD is verified in the submission system and will appear in the published article. PLOS supports the use of ORCID, and we encourage all coauthors to register for an ORCID iD and use it as well. Please encourage your coauthors to verify their ORCID iD within the submission system before final acceptance, as unverified ORCID iDs will not appear in the published article. Only the individual author can complete the verification step; PLOS staff cannot verify ORCID iDs on behalf of authors.

We look forward to receiving your revised manuscript.

Kind regards,

Yamir Moreno

Academic Editor

PLOS Computational Biology

Jennifer Flegg

Section Editor

PLOS Computational Biology

**Additional Editor Comments (if provided):**

**Journal Requirements:**

If the reviewer comments include a recommendation to cite specific previously published works, please review and evaluate these publications to determine whether they are relevant and should be cited. There is no requirement to cite these works unless the editor has indicated otherwise.

1) Thank you for stating "Data and code to replicate this study can be found below on Harvard Dataverse https://doi.org/10.7910/DVN/A1BUUD"

We strongly recommend all authors deposit their data before acceptance, as the process can be lengthy and hold up publication timelines. Please note that, though access restrictions are acceptable now, your entire minimal dataset will need to be made freely accessible if your manuscript is accepted for publication. This policy applies to all data except where public deposition would breach compliance with the protocol approved by your research ethics board. If you are unable to adhere to our open data policy, please kindly revise your statement to explain your reasoning and we will seek the editor's input on an exemption.

**Reviewers' comments:**

Reviewer's Responses to Questions

**Comments to the Authors:**

**Please note here if the review is uploaded as an attachment.**

Reviewer #1: I think the authors have quite satisfactorily amended the major issues flagged in the first round of revision. I sinsceresly congratulate them as I find now the manuscript ready for publication.

Reviewer #2: I thank the authors for the detailed revisions which greatly improve the quality of the manuscript and clarify a number of points that I had questions on. In particular, I think the added text around the Tolbit regression was very helpful and the new analysis of the 3 phases of vaccine uptake strengthened the point. In addition, the role of corrections to reported vaccine data in Fig. 1 was clarifying.

I have two points (both straightforward) that I think need to be addressed in the final version of the paper:

1. I appreciate the authors presenting the decomposition of all 51 states and regions in Fig. A.2. However, it appears that the true weekly vaccinations were never added to the plots. In all 51 plots, there is a solid black line a 0, which would suggest that there was no weekly vaccination for any of the states.

2. The author summary starts with "Why do some communities vaccinate quickly while others lag behind—even when vaccines are equally available?" I think this is a very interesting and catchy question, and certainly the authors method (through the decomposition) could address this. However, besides just plotting the individual decomposition curves in Fig. 6C-E and Fig. A.2, the authors do not really address what is different between the different states. I think therefore the authors should either change their motivating question to reflect the results they investigate in detail (e.g., the 3 different phases) or they should add more analysis to what differs between the different states (e.g., the authors compute the correlations of each component of the decomposition for all states with time).

**********

**Have the authors made all data and (if applicable) computational code underlying the findings in their manuscript fully available?**

The PLOS Data policy requires authors to make all data and code underlying the findings described in their manuscript fully available without restriction, with rare exception (please refer to the Data Availability Statement in the manuscript PDF file). The data and code should be provided as part of the manuscript or its supporting information, or deposited to a public repository. For example, in addition to summary statistics, the data points behind means, medians and variance measures should be available. If there are restrictions on publicly sharing data or code —e.g. participant privacy or use of data from a third party—those must be specified.

Reviewer #1: Yes

Reviewer #2: None

**********

PLOS authors have the option to publish the peer review history of their article (what does this mean?). If published, this will include your full peer review and any attached files.

If you choose “no”, your identity will remain anonymous but your review may still be made public.

**Do you want your identity to be public for this peer review?**  For information about this choice, including consent withdrawal, please see our Privacy Policy.

Reviewer #1: No

Reviewer #2: **Yes:** William Redman

[NOTE: If reviewer comments were submitted as an attachment file, they will be attached to this email and accessible via the submission site. Please log into your account, locate the manuscript record, and check for the action link "View Attachments". If this link does not appear, there are no attachment files.]

**Figure resubmission:**

-->While revising your submission, we strongly recommend that you use PLOS’s NAAS tool (https://ngplosjournals.pagemajik.ai/artanalysis) to test your figure files. NAAS can convert your figure files to the TIFF file type and meet basic requirements (such as print size, resolution), or provide you with a report on issues that do not meet our requirements and that NAAS cannot fix.

After uploading your figures to PLOS’s NAAS tool - https://ngplosjournals.pagemajik.ai/artanalysis, NAAS will process the files provided and display the results in the "Uploaded Files" section of the page as the processing is complete. If the uploaded figures meet our requirements (or NAAS is able to fix the files to meet our requirements), the figure will be marked as "fixed" above. If NAAS is unable to fix the files, a red "failed" label will appear above. When NAAS has confirmed that the figure files meet our requirements, please download the file via the download option, and include these NAAS processed figure files when submitting your revised manuscript.

**Reproducibility:**

To enhance the reproducibility of your results, we recommend that authors of applicable studies deposit laboratory protocols in protocols.io, where a protocol can be assigned its own identifier (DOI) such that it can be cited independently in the future. Additionally, PLOS ONE offers an option to publish peer-reviewed clinical study protocols. Read more information on sharing protocols at https://plos.org/protocols?utm_medium=editorial-email&utm_source=authorletters&utm_campaign=protocols

# Author comment: Author response to Decision Letter 2

**DOI:** 10.1371/journal.pcbi.1013988.r004

12 Jun 2026

Attachment Submitted filename: author_response_auresp_2.docx

# Peer review: Decision Letter 2

**DOI:** 10.1371/journal.pcbi.1013988.r005

**Authors:** Yamir Moreno, Jennifer Flegg

29 Jun 2026

Dear Dr. Xu,

We are pleased to inform you that your manuscript 'Population-Level Behavioral and Structural Drivers of COVID-19 Vaccine Uptake in the US' has been provisionally accepted for publication in PLOS Computational Biology.

Before your manuscript can be formally accepted you will need to complete some formatting changes, which you will receive in a follow up email. A member of our team will be in touch with a set of requests.

Please note that your manuscript will not be scheduled for publication until you have made the required changes, so a swift response is appreciated.

IMPORTANT: The editorial review process is now complete. PLOS will only permit corrections to spelling, formatting or significant scientific errors from this point onwards. Requests for major changes, or any which affect the scientific understanding of your work, will cause delays to the publication date of your manuscript.

Should you, your institution's press office or the journal office choose to press release your paper, you will automatically be opted out of early publication. We ask that you notify us now if you or your institution is planning to press release the article. All press must be co-ordinated with PLOS.

Thank you again for supporting Open Access publishing; we are looking forward to publishing your work in PLOS Computational Biology.

Best regards,

Yamir Moreno

Academic Editor

PLOS Computational Biology

Jennifer Flegg

Section Editor

PLOS Computational Biology

***********************************************************

Reviewer's Responses to Questions

**Comments to the Authors:**

**Please note here if the review is uploaded as an attachment.**

Reviewer #2: The authors have addressed all of my questions. I thank them for their updates and their work!

**********

**Have the authors made all data and (if applicable) computational code underlying the findings in their manuscript fully available?**

The PLOS Data policy requires authors to make all data and code underlying the findings described in their manuscript fully available without restriction, with rare exception (please refer to the Data Availability Statement in the manuscript PDF file). The data and code should be provided as part of the manuscript or its supporting information, or deposited to a public repository. For example, in addition to summary statistics, the data points behind means, medians and variance measures should be available. If there are restrictions on publicly sharing data or code —e.g. participant privacy or use of data from a third party—those must be specified.

Reviewer #2: Yes

**********

PLOS authors have the option to publish the peer review history of their article (what does this mean?). If published, this will include your full peer review and any attached files.

If you choose “no”, your identity will remain anonymous but your review may still be made public.

**Do you want your identity to be public for this peer review?**  For information about this choice, including consent withdrawal, please see our Privacy Policy.

Reviewer #2: **Yes:** William Redman

# Editor report: Acceptance letter

**DOI:** 10.1371/journal.pcbi.1013988.r006

**Authors:** Yamir Moreno, Jennifer Flegg

PCOMPBIOL-D-26-00285R2

Population-Level Behavioral and Structural Drivers of COVID-19 Vaccine Uptake in the US

Dear Dr Ghaffarzadegan,

I am pleased to inform you that your manuscript has been formally accepted for publication in PLOS Computational Biology. Your manuscript is now with our production department and you will be notified of the publication date in due course.

The corresponding author will soon be receiving a typeset proof for review, to ensure errors have not been introduced during production. Please review the PDF proof of your manuscript carefully, as this is the last chance to correct any errors. Please note that major changes, or those which affect the scientific understanding of the work, will likely cause delays to the publication date of your manuscript.

Soon after your final files are uploaded, unless you have opted out, the early version of your manuscript will be published online. The date of the early version will be your article's publication date. The final article will be published to the same URL, and all versions of the paper will be accessible to readers.

For Research, Software, and Methods articles, you will receive an invoice from PLOS for your publication fee after your manuscript has reached the completed accept phase. If you receive an email requesting payment before acceptance or for any other service, this may be a phishing scheme. Learn how to identify phishing emails and protect your accounts at https://explore.plos.org/phishing.

Thank you again for supporting PLOS Computational Biology and open-access publishing. We are looking forward to publishing your work!

With kind regards,

Anitha Samidurai

PLOS Computational Biology | Carlyle House, Carlyle Road, Cambridge CB4 3DN | United Kingdom ploscompbiol@plos.org | Phone +44 (0) 1223-442824 | ploscompbiol.org | @PLOSCompBiol
